# Impaired innate and adaptive immune responses to BNT162b2 SARS-CoV-2 vaccination in systemic lupus erythematosus

**DOI:** 10.1172/jci.insight.176556

**Published:** 2024-03-08

**Authors:** Kavita Y. Sarin, Hong Zheng, Yashaar Chaichian, Prabhu S. Arunachalam, Gayathri Swaminathan, Alec Eschholz, Fei Gao, Oliver F. Wirz, Brandon Lam, Emily Yang, Lori W. Lee, Allan Feng, Matthew A. Lewis, Janice Lin, Holden T. Maecker, Scott D. Boyd, Mark M. Davis, Kari C. Nadeau, Bali Pulendran, Purvesh Khatri, Paul J. Utz, Lisa C. Zaba

**Affiliations:** 1 Department of Dermatology,; 2 Institute for Immunity, Transplantation and Infection,; 3 Center for Biomedical Informatics Research, Department of Medicine, School of Medicine, and; 4 Department of Medicine, Division of Immunology and Rheumatology, Stanford University, Stanford, California, USA.; 5 Department of Immunobiology, University of Arizona, Tucson, Arizona, USA.; 6 Department of Pathology and; 7 Department of Pediatrics, Division of Pediatric Pulmonary Medicine, Stanford University School of Medicine, Stanford, California, USA.; 8 Department of Microbiology and Immunology, Howard Hughes Medical Institute, Stanford University, Stanford, California, USA.; 9 Department of Environmental Gealth, Harvard T.H. Chan School of Public Health, Boston, Massachusetts, USA.; 10 Department of Microbiology and Immunology, Stanford University School of Medicine, Stanford University, Stanford, California, USA.

**Keywords:** Autoimmunity, Vaccines, Adaptive immunity, Innate immunity

## Abstract

Understanding the immune responses to SARS-CoV-2 vaccination is critical to optimizing vaccination strategies for individuals with autoimmune diseases, such as systemic lupus erythematosus (SLE). Here, we comprehensively analyzed innate and adaptive immune responses in 19 patients with SLE receiving a complete 2-dose Pfizer-BioNTech mRNA vaccine (BNT162b2) regimen compared with a control cohort of 56 healthy control (HC) volunteers. Patients with SLE exhibited impaired neutralizing antibody production and antigen-specific CD4*^+^* and CD8*^+^* T cell responses relative to HC. Interestingly, antibody responses were only altered in patients with SLE treated with immunosuppressive therapies, whereas impairment of antigen-specific CD4*^+^* and CD8*^+^* T cell numbers was independent of medication. Patients with SLE also displayed reduced levels of circulating CXC motif chemokine ligands, CXCL9, CXCL10, CXCL11, and IFN-γ after secondary vaccination as well as downregulation of gene expression pathways indicative of compromised innate immune responses. Single-cell RNA-Seq analysis reveals that patients with SLE showed reduced levels of a vaccine-inducible monocyte population characterized by overexpression of IFN-response transcription factors. Thus, although 2 doses of BNT162b2 induced relatively robust immune responses in patients with SLE, our data demonstrate impairment of both innate and adaptive immune responses relative to HC, highlighting a need for population-specific vaccination studies.

## Abstract 2

SARS-CoV-2 mRNA vaccination reduces innate, T cell, and B cell immune responses in patients with systemic lupus erythematosus, highlighting a need for population-specific vaccination studies.

## Introduction

Systemic lupus erythematosus (SLE) is a multiorgan, heterogeneous autoimmune disease (AID) associated with aberrant innate and adaptive immune function (1, 2). Persons with SLE have up to a 3-fold higher risk of COVID-19–related infection and hospitalization than those without SLE, likely due to a combination of intrinsic immune dysfunction, immunosuppressive treatment, and lupus-related comorbidities (3–6). Therefore, vaccination is an important component in the standard of care for patients with SLE. The mRNA vaccines directed against SARS-CoV-2 Spike protein are highly effective in preventing SARS-CoV-2 infection, with an efficacy rate as high as 94% in healthy adults. However, the initial phase III trials excluded immunocompromised patients, such as those with AID or individuals treated with immunosuppressive agents. Consequently, less is known about the efficacy of SARS-CoV-2 vaccination in patients with an AID, including those with SLE (7, 8). Additionally, some patients with SLE are hesitant to undergo immunization due to concerns about disease exacerbation after vaccination (9–12). Thus, there is a need for a comprehensive characterization of the immune responses to SARS-CoV-2 vaccines in patients with SLE to determine the optimal vaccination strategies for these at-risk individuals.

Here, we conducted a “systems vaccinology” study of the 2-dose Pfizer-BioNTech (BNT162b2) COVID-19 mRNA vaccine in 19 individuals with SLE and compared their responses with healthy controls (HC) at baseline and following vaccination until 3 weeks after the second dose. Comprehensive study assessments included measures of safety, such as SLE disease activity indices, quantitation of autoantibodies, and anti-cytokine antibodies using custom microbead antigen arrays. We assessed humoral immunity by measuring IgG antibodies to the Spike protein receptor-binding domain (RBD) and virus-neutralizing antibodies to 3 SARS-CoV-2 virus strains. Antigen-specific T cell reactivity was also analyzed using major histocompatibility complex (MHC) Class I and Class II spheromers loaded with peptides derived from SARS-CoV-2 Spike protein. Finally, we performed bulk transcriptome sequencing, Olink proteomics, and single-cell profiling to elucidate changes in blood cell transcriptomes, cytokine/chemokine activation, and innate/adaptive immune cell pathways, in response to vaccination. Results were integrated and further stratified by medication use to determine the effect of medications on vaccine responsiveness.

## Results

### Patient population and demographics.

**Figure 1:**
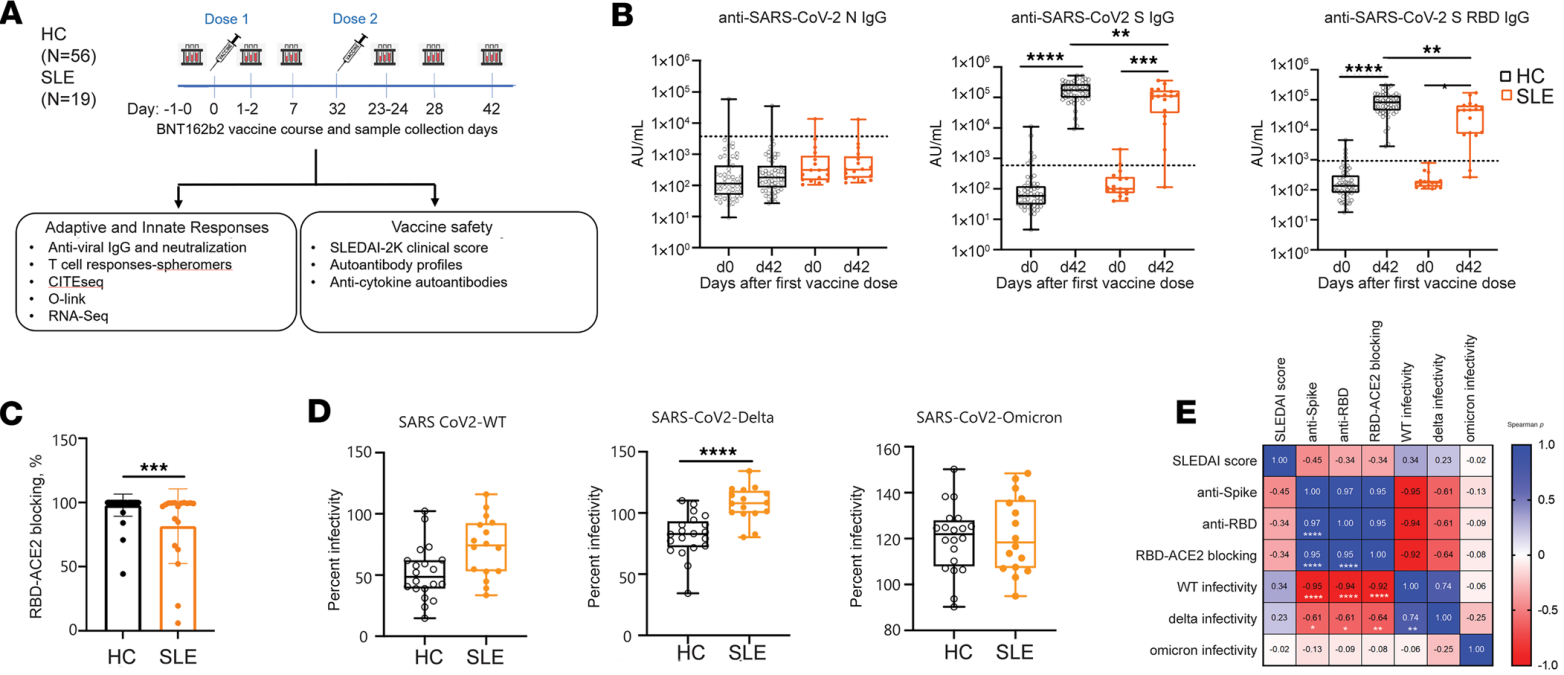
Longitudinal monitoring of BNT162b2 vaccine elicited protective humoral responses to SARS-CoV-2 after vaccination in patients with SLE and in HC. (**A**) Study design, samples collected, and analysis of immune responses to BNT162b2 vaccine. (**B**) Anti–SARS-CoV-2 N, Spike (S), and anti-RBD antibodies in fully vaccinated patients with SLE compared with HC was assessed by Meso Scale Diagnostics multiplex analysis of patient plasma (day 0 versus day 42). (**C**) Serum from vaccinated patients with SLE (day 42) show impaired ACE2-RBD blocking capability. (**D**) Serum from fully vaccinated patients with SLE (day 42) is less efficient at neutralizing SARS-CoV-2 strains, as indicated, compared with HC in a pseudovirus neutralization assay. (**E**) Spearman correlation analysis of BNT162b2-elicited antiviral humoral response in patients with SLE and SLE disease score (SLEDAI). Data are shown as mean ± SD; *n* = 53 HC, 18 SLE for **B**. One-way ANOVA with Bonferroni post hoc test; *n* = 53 HC, 18 SLE for **C**. *n* = 20 HC, 16 SLE for **D**. Data are shown as mean ± SD. Students *t* test, 2-tailed. *P < 0.05, **P < 0.01, ***P < 0.001, ****P < 0.0001.

From March 2021 to May 2021, 19 patients at Stanford Hospital and Clinics with a diagnosis of SLE as defined by fulfillment of the 2019 European Alliance of Associations for Rheumatology (EULAR)/American College of Rheumatology (ACR) Classification Criteria (13) and stable disease were enrolled in this study (Table 1 and Supplemental Table 1; supplemental material available online with this article; https://doi.org/10.1172/jci.insight.176556DS1). Stable disease required the following: baseline Systemic Lupus Erythematosus Disease Activity Index 2000 (SLEDAI-2K) < 10 and clinical SLEDAI-2K < 8, no change in systemic immunosuppression for at least 2 months preceding the baseline visit, no disease flare at the baseline visit (defined as ≥ 4-point increase in SLEDAI-2K plus signs/symptoms of SLE necessitating an increase in immunosuppression), and absence of active nephritis or active CNS involvement (14). Upon study entry, prednisone dose was required to be < 25 mg/day, and patients who had received > 40 mg/day of prednisone equivalent within 2 months of the baseline visit were excluded. The HC cohort was enrolled between December 2020 and February 2021 and has been previously published (15). Patients in both cohorts received 2 doses of the BNT162b2 on day 0 and day 21 and were followed with serial clinical and laboratory assessments until day 42 (Figure 1A).

### Vaccinated patients with SLE show reduced SARS-CoV-2 humoral responses compared with HC.

We first measured IgG antibodies specific to the Spike protein and RBD of SARS-CoV-2 at baseline, after primary vaccination (days 1 or 2 and 7), and after secondary vaccination (days 23 or 24, 28, and 42). Although the majority of participants in the SLE and HC cohorts mounted robust antibody responses after 2-dose vaccination with BNT162b2, a subset of patients with SLE displayed reduced levels of IgG antibodies against SARS-CoV-2 Spike and RBD at day 42 relative to HC (Figure 1B and Supplemental Figure 1A; *P* < 0.01, 2-way ANOVA). In addition, pseudoneutralization and angiotensin-converting enzyme 2–RBD (ACE2-RBD) blocking assays revealed significantly lower neutralizing ability in serum derived from patients with SLE (as indicated by blocking of the ACE2-RBD interaction) compared with HC, suggesting decreased humoral protection against SARS-CoV-2 infection (Figure 1C; *P* < 0.0001, 2-way ANOVA). Increased antibodies against seasonal coronavirus were not detected after vaccination in either cohort, consistent with prior studies (Supplemental Figure 1B) (16).

We next measured the ability of serum samples to neutralize 3 common strains of SARS-CoV-2: WT, Delta, and Omicron (BA1). Patients with SLE and HC displayed neutralizing activity against all 3 strains of SARS-CoV-2. However, neutralizing activity was significantly impaired for patients with SLE relative to HC for the SARS-CoV-2–Delta strain (Figure 1D; mean infectivity of 107.5% in patients with SLE versus 81.8% in HC; *P* < 0.0001, Kolmogorov-Smirnov). Moreover, there was a trend toward impaired neutralization activity against the SARS-CoV-2 WT strain (mean infectivity of 72.0 in patients with SLE versus 57.7 in HC; *P* = 0.10, Kolmogorov-Smirnov). However, no significant difference in neutralization activity was observed for the Omicron BA1 strain, likely due to a general reduction of vaccine effectiveness against SARS-CoV-2 in HC (17). As expected, we observed a strong correlation between induction of IgG antibodies against both Spike and RBD and inhibition of binding between ACE2 and the SARS-CoV-2 RBD (i.e., RBD-ACE2 blocking antibodies) (Figure 1E and Supplemental Figure 1D). IgG antibody levels were also inversely correlated with SARS-CoV-2 WT and Delta infectivity in neutralization assays (Figure 1E). Taken together, these data suggest that some patients with SLE show impaired humoral responses following vaccination with BNT162b2, which may increase their susceptibility to SARS-CoV-2 infection.

### Immunosuppressive agents contribute to reduced vaccine efficacy in patients with SLE.

Use of immunosuppressive treatments, such as methotrexate (MTX) and mycophenolate mofetil (MMF), is thought to be a major factor affecting vaccine response against SARS-CoV-2 (18). To determine the effect of immunosuppressive medications on vaccine efficacy in patients with SLE, we stratified our SLE cohort into 3 groups — hydroxychloroquine (HCQ) only; HCQ plus an immunosuppressive conventional synthetic disease-modifying antirheumatic drug (csDMARD) such as MTX or MMF; or HCQ plus a biologic DMARD (bDMARD), such as belimumab or rituximab — and compared the Spike-RBD neutralizing IgG antibody response among the different groups. We observed a reduced neutralizing antibody response in patients with SLE treated with a csDMARD and/or a bDMARD compared with those taking HCQ alone (Supplemental Figure 1C), demonstrating a strong effect of immunosuppressive medications on vaccine efficacy. Consistent with this observation, the lone patient with SLE (patient #28) not taking any medication developed a neutralizing activity response equivalent to that observed in HC after 2-dose SARS-CoV-2 vaccination, whereas the only patient with SLE taking both a csDMARD and bDMARD (patient #0012) developed virtually no neutralizing activity after vaccination.

### Patients with SLE with autoantibodies against Ro52 and Ro60 are less responsive to vaccination.

**Figure 2:**
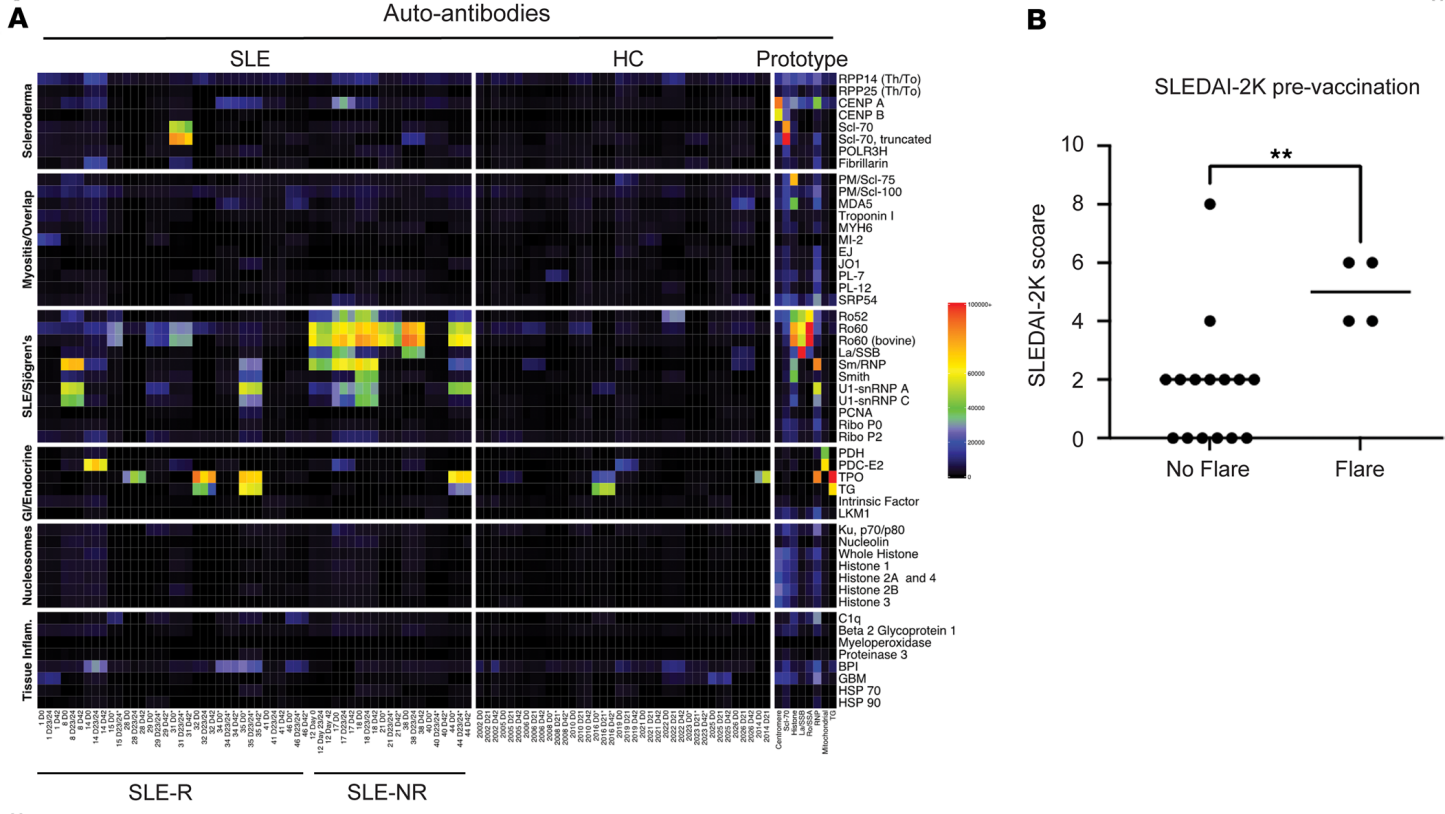
BNT162b2 mRNA vaccination does not induce new autoantibodies in patients with SLE. (**A**) Heatmap of autoantibody levels in serum of patients with SLE (R, responders; NR, nonresponders) and healthy controls (HC) on indicated days after vaccination was measured using a 51-plex connective tissue disease (CTD) antigen array using a microbead assay. MFI values are shown. *n* = 13 HC, 19 SLE. (**B**) SLE disease flare requiring treatment change after BNT162b2 vaccine administration. Unpaired 2-tailed Students *t* test; **P < 0.01.

To better evaluate vaccine responsiveness in our study cohorts, we defined all patients who mounted a neutralizing ACE-RBD response equivalent to that observed in HC at day 42 as responders, and we defined those with a diminished response — i.e., 2 SDs below the mean of the HC cohort — as nonresponders (NRs). The number of NRs in the SLE cohort (7 of 19) was significantly greater than in the HC cohort (3 of 56; *P* = 0.0013, Fisher’s exact test). Interestingly, within the SLE cohort, we further found that NRs were more likely to have anti-Ro52 and anti-Ro60 IgG autoantibodies than responders (6 of 7 NR versus 0 of the 11 responders; *P* = 0.0004, Fisher’s exact test) (Figure 2A). However, SLE NRs did not differ significantly from responders for other autoantibodies, including anti-nuclear antibody (ANA) and anti–double stranded DNA, anti-Smith, and anti-IFN antibodies.

### Neither induction of de novo autoantibodies nor major adverse reactions were observed in patients with SLE during the 42-day study.

Prior studies have identified low incidences of de novo serum autoantibody formation after 3-dose SARS-CoV-2 vaccination (19–21). A subset of hospitalized COVID patients develop new autoantibodies after SARS-CoV-2 infection (22). SLE patients may be particularly primed toward development of increased levels of existing autoantibodies or production of entirely new autoantibodies after vaccination. To determine whether vaccination could induce autoantibody formation in patients with SLE, we characterized autoantibody profiles in 19 patients with SLE and 13 HC at days 0, 23 or 24, and 42 using a 51-plex connective tissue disease antigen array in combination with a 56-plex cytokine array to measure anti-cytokine antibodies. As reported previously in HC and in a cystic fibrosis cohort, BNT162b2 vaccination was not associated with generation of new autoantibodies or an increase in existing autoantibody titers in any patients with SLE or HC (Figure 2A). Additionally, with the exception of 1 patient with SLE who developed transient elevation of preexisting anti–IFN-γ autoantibodies, we did not detect any new anti-cytokine antibodies or increased levels of existing antibodies (Supplemental Figures 2 and 3). Consistent with these findings, we observed no major adverse events in patients with SLE or HC after BNT162b2 vaccination. However, 4 of 19 participants with SLE experienced a minor flare. Three patients experienced musculoskeletal symptoms, and 1 patient developed a flare of her malar rash. Each flare responded to a short low-dose prednisone taper (Figure 2B).

### Patients with SLE produce reduced levels of SARS-CoV-2 Spike–specific CD8^+^ and CD4^+^ T cells after 2-dose BNT162b2 vaccination.

**Figure 3:**
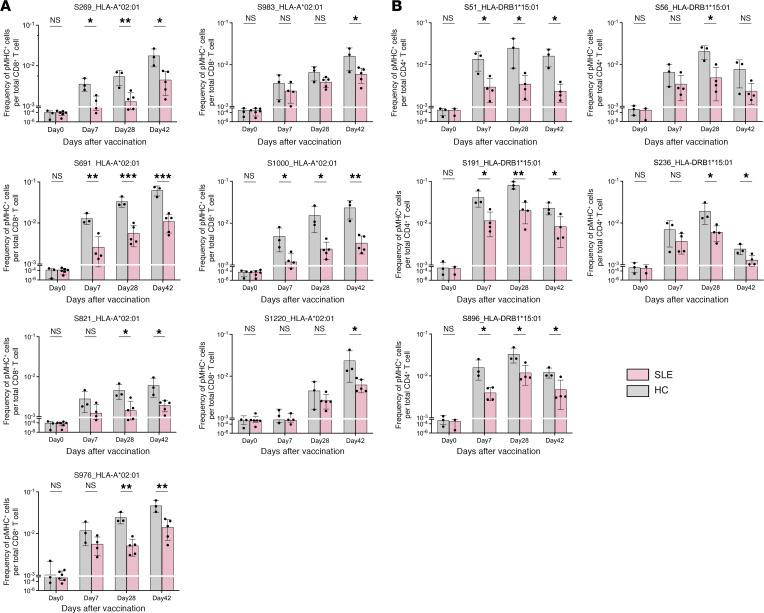
Fully vaccinated patients with SLE have markedly reduced frequencies of SARS-CoV-2 Spike–specific CD8^+^ and CD4^+^ T cells. (**A**) SARS-CoV-2 epitope–specific CD8^+^ T cell frequencies in PBMC isolated from HLA-A*02:01 patients with SLE and healthy controls (HC) at indicated time points after BNT162b2 administration was assessed using pMHC spheromer displaying SARS-CoV-2 epitopes. *n* = 3 HC, *n* = 6 SLE. (**B**) SARS-CoV-2 epitope–specific CD4^+^ T cell frequencies in PBMC isolated from HLA-DRB1*15:01 patients with SLE and HC at indicated time points after BNT162b2 administration was assessed using pMHC spheromer displaying SARS-CoV-2 epitopes. *n* = 3 HC, *n* = 4 SLE. Data are shown as mean ± SD. *P* values were determined by unpaired multiple *t* test (0.05 > *P > 0.01; 0.01 > **P > 0.001; ***P < 0.001).

Recent studies have shown that SARS-CoV-2 vaccination can induce durable antigen-specific T cell responses in healthy individuals (15, 23, 24). We therefore evaluated antigen-specific T cell responses in 10 patients with SLE (*n* = 6 HLA-A*02:01, *n* = 4 HLA-DRB1*15:01) and 6 HC (*n* = 3 HLA-A*02:01, *n* = 3 HLA-DRB1*15:01) using peptide MHC (pMHC) spheromers displaying SARS-CoV-2 Spike epitopes (24). Results show that all patients with SLE had a lower frequency of CD8^+^ and CD4^+^ Spike–specific T cells than HC throughout the vaccination series (Figure 3, A and B; *P* < 0.05 by Mann-Whitney *U* test). Unlike the antibody response, the T cell response appears independent of medications and, thus, may represent an intrinsic aspect of the disease (Supplemental Figure 4).

### Higher baseline levels of type I IFN signaling and innate immune pathway activation are associated with lower vaccine efficacy in patients with SLE.

**Figure 4:**
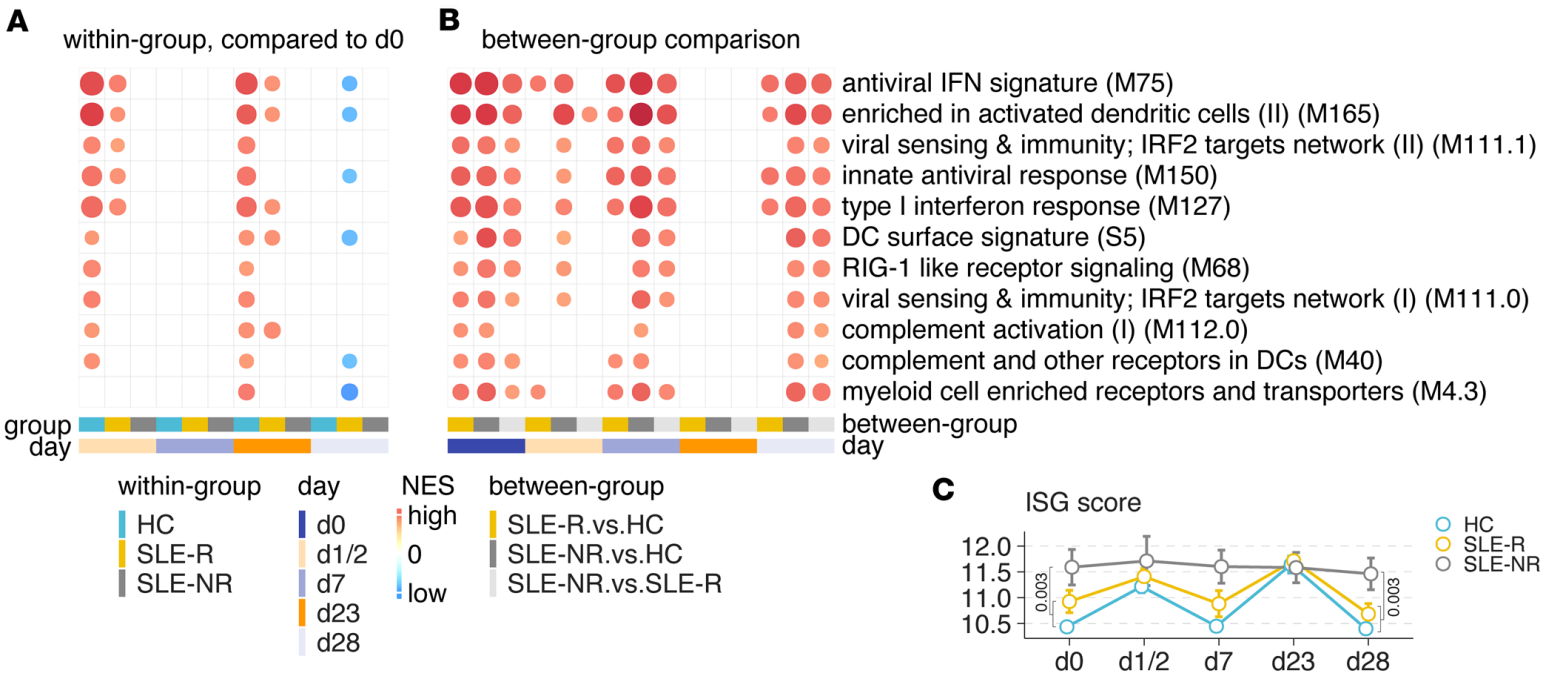
Temporal changes in whole blood transcriptional profiles of patients with SLE after BNT162b2 vaccination. (**A**) Gene set enrichment analysis of blood transcriptome modules (BTMs) enriched at each time point compared with baseline (day 0). The BTM terms with FDR less than 5% were shown as circles. NES, normalized enrichment score. (**B**) Gene set enrichment of SLE vaccine responders (SLE-R) compared with healthy controls (HC), SLE vaccine nonresponders (SLE-NR) compared with HC, and SLE-NR compared with SLE-R. The BTM terms with FDR less than 5% were shown as circles. (**C**) ISG score for each cohort across time points. The *P* values for day 0 (SLE-NR, *n* = 7; SLE-R, *n* = 11; HC:,*n* = 31) and day 28 (SLE-NR, *n* = 7; SLE-R, *n* = 11; HC, *n* = 30) were calculated using 1-sided Wilcoxon rank-sum test.

To assess transcriptional changes after vaccination, we performed bulk RNA-Seq of whole blood from 18 patients with SLE and compared the transcriptomic data to existing data from 32 HC (15). Gene set enrichment analysis (GSEA) revealed increased antiviral IFN response 1 or 2 days after primary or secondary vaccination in HC and SLE vaccine responders (Figure 4A). GSEA revealed baseline increases in innate immune pathways — including type I IFN signaling, RIG-I–like receptor signaling, and antiviral signatures — in the SLE cohort compared with HC (Figure 4B). Interestingly, within the SLE cohort, vaccine NRs showed increased enrichment of innate immunity pathways, type I IFN signaling, and RIG-I–like receptor signaling genes at baseline and after vaccination relative to responders (Figure 4B). We further computed an IFN-stimulated gene (ISG) score from genes in enriched pathways involved in IFN response and showed that the ISG score in SLE vaccine NRs was higher than HC and SLE vaccine responders both at baseline and after vaccination (Figure 4C and Supplemental Figure 5A). These results suggest that elevated baseline type I IFN signaling is associated with reduced antibody response after vaccination.

### Deconvolution of bulk sequencing data reveals increased CD14^+^ monocytes in patients with SLE.

We next performed deconvolution of our bulk transcriptome data to assess cellular composition changes in patients with SLE and HC after vaccination using immunoStates, as described previously (25). Consistent with markedly reduced Spike-specific CD4^+^ and CD8^+^ T cell frequencies in PBMCs isolated from patients with SLE in our Spheromer assay, our analysis revealed reduced T cell proportions in patients with SLE compared with HC. Patients with SLE also displayed increased numbers of CD14^+^ monocytes consistent with the enriched innate immune pathway signatures in patients with SLE compared with HC. Notably, vaccine NRs in the SLE cohort had the highest proportion of CD14^+^ monocytes and lowest T cell proportions at baseline, compared with either HC or SLE-cohort vaccine responders (Supplemental Figure 5B).

### Higher baseline levels of type I IFN signaling and innate immune pathway activation are associated with lower vaccine efficacy in patients with SLE.

**Figure 5:**
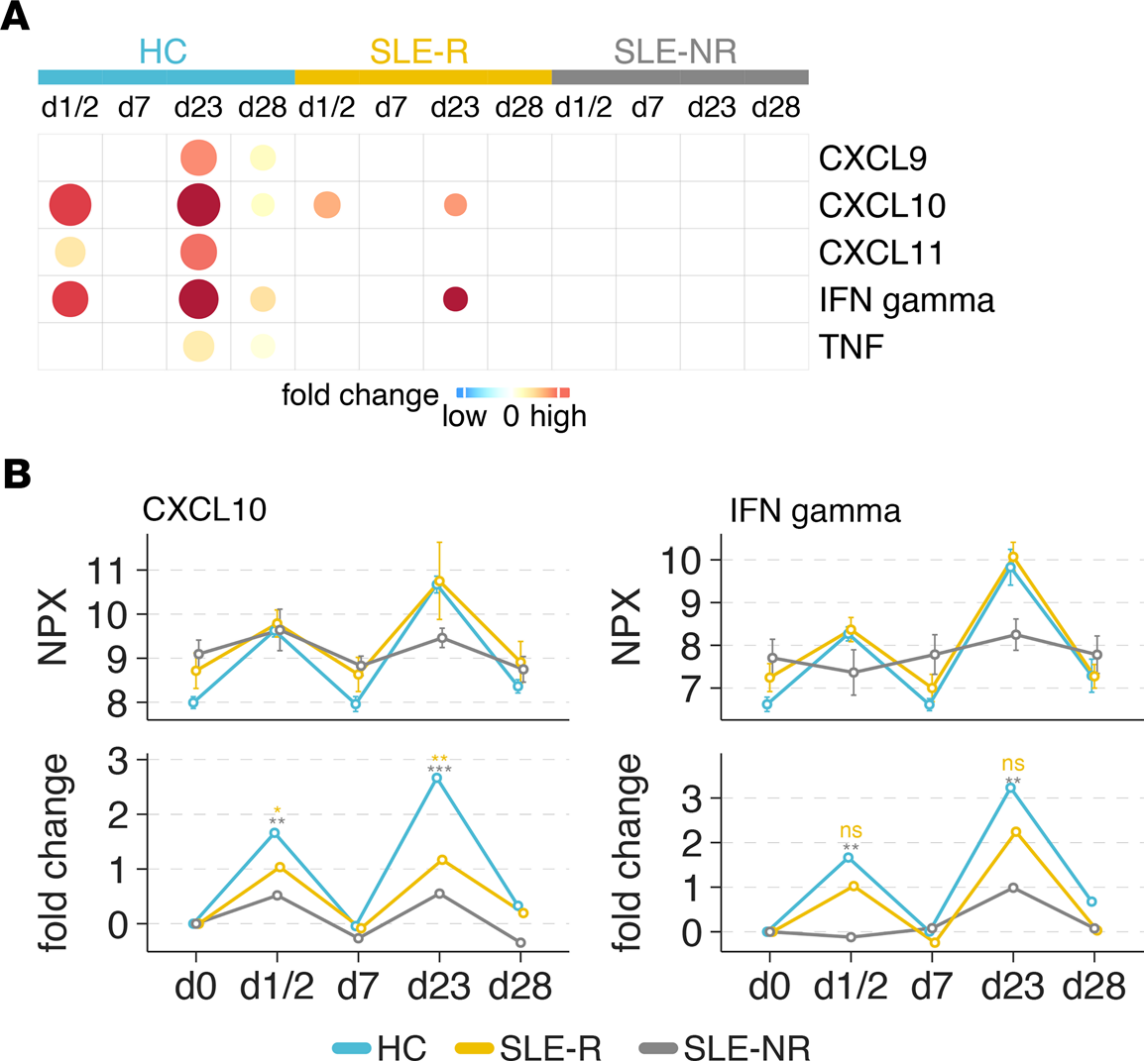
Cytokine and chemokine responses in patients with SLE and healthy controls (HC) after BNT162b2 vaccination. Levels of indicated cytokines and chemokines were measured in plasma of patients with SLE and HC using Olink Target 96 inflammation panel. (**A**) Heatmap demonstrating fold change in CXCL9, CXCL10, CXCL11, IFN-γ, and TNF plasma levels compared with baseline (day 0). The fold changes with FDR less than 20% were shown on the heatmap. (**B**) Line graphs depicting normalized protein expression (NPX) and fold change of CXCL10 and IFN-γ. The unadjusted *P* values were from between-group limma analysis after adjusting for baseline. * *P* < 0.05, ** *P* < 0.01, ***P < 0.001. Yellow asterisks are the comparisons between SLE-R and HC; gray asterisks are the comparisons between SLE-NR and HC. Samples numbers of each group: day 0 (SLE-NR, *n* = 7; SLE-R, *n* = 11; HC, *n* = 31); day 1/2 (SLE-NR, *n* = 5; SLE-R, *n* = 10; HC, *n* = 31); day 23 (SLE-NR, *n* = 4; SLE-R, *n* = 3; HC, *n* = 10).

To further investigate the effect of BNT162b2 vaccination on the plasma proteome, we measured cytokine and chemokine levels in the plasma of 18 patients with SLE at serial time points after primary and secondary vaccination using the Olink Target 96 inflammation panel and compared the results to existing Olink data from 31 HC. Consistent with our bulk RNA-Seq data, patients with SLE also showed elevated levels of CXCL10 at baseline compared with HC (Supplemental Figure 6A). In addition, patients with SLE displayed strikingly decreased induction of CXCL9, CXCL10, CXCL11, TNF, and IFN-γ compared with HC following vaccination (Figure 5, A and B, and Supplemental Figure 6B). Moreover, although impaired induction of CXCL9, CXCL10, CXCL11, TNF, and IFN-γ was detected in both NRs and responders in the SLE cohort, differences were most substantial in the NR group (Figure 5, A and B, and Supplemental Figure 6B).

### IFN-γ–associated myeloid cells are increased in SLE but not induced by BNT162b2 vaccination.

**Figure 6:**
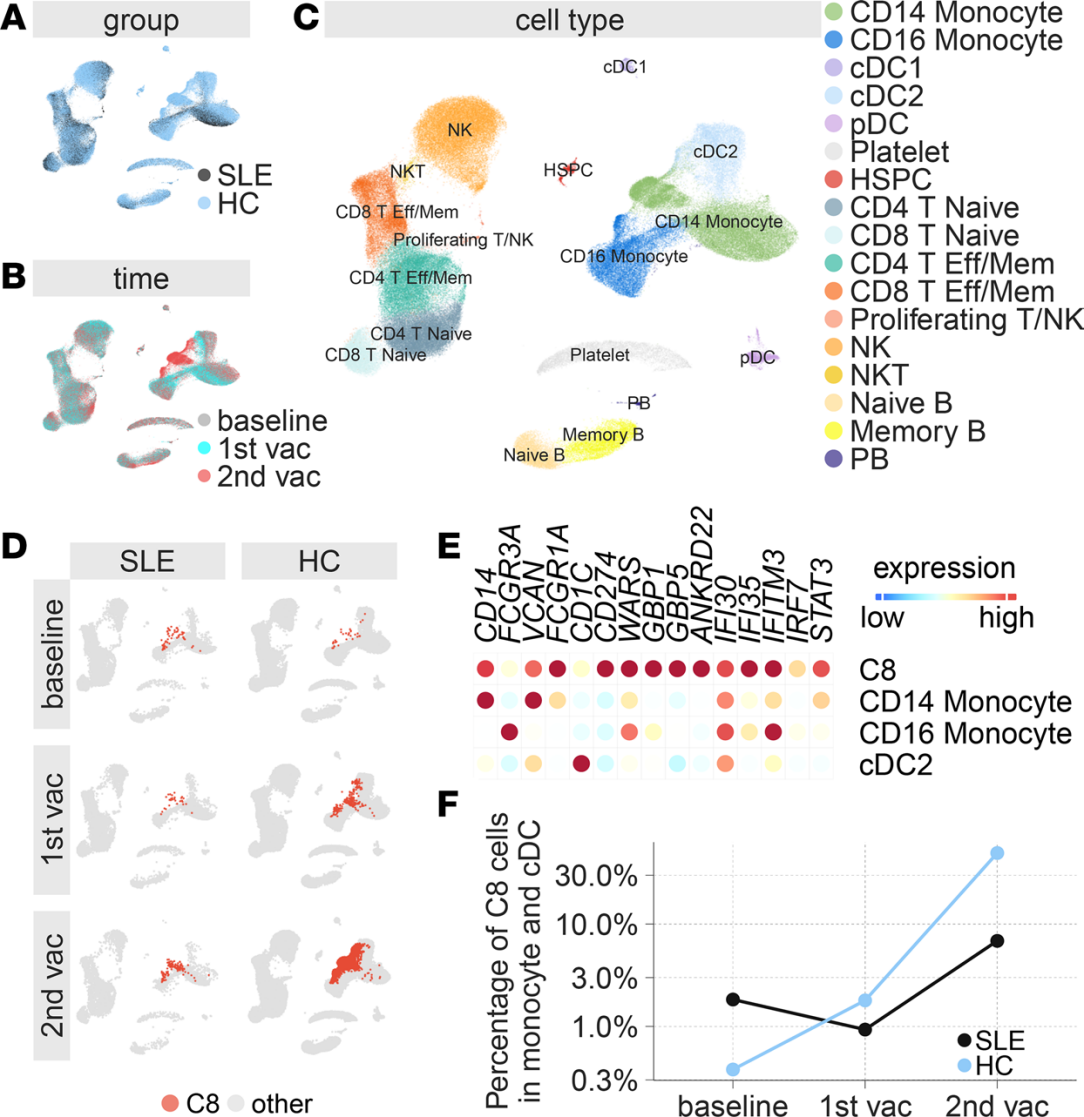
Single-cell transcriptional response after primary and secondary vaccination in patients with SLE and healthy controls (HC). (**A**) UMAP representation of peripheral mononuclear cells in patients with SLE and in HC. (**B**) UMAP representation of peripheral mononuclear cells at baseline, 1 or 2 days after primary vaccination, and 1 or 2 days after secondary vaccination. (**C**) UMAP representation of cell types identified by single-cell transcriptional profiling. (**D**) Feature plots across time points showing C8 cluster in red at baseline and after primary and secondary vaccination. (**E**) Heatmap of the mean expression of myeloid cell markers and genes highly expressed in C8 cluster. Rows show the C8 cluster and the remaining monocyte and DC subsets after separating out the C8 cells. A full heatmap showing all cell types is shown in Supplemental Figure 7B. (**F**) Percentage of C8 cluster cells in monocytes and cDC cells in patients with SLE (black) and in HC (blue). The χ^2^ test was performed comparing the cell proportions of C8 cluster out of total monocytes and cDCs from 3 patients with SLE and 6 HC at baseline (1.8% versus 0.038%, *P* = 4.2 × 10^–15^) and after secondary vaccination (6.9 % versus 49.4%, *P* = 2.2 × 10^–16^).

The striking differences in the magnitude of effector immune responses (humoral and cellular) as well as in the frequencies of T cell and CD14^+^ monocyte cell population in our SLE cohort led us to further characterize the changes in immune cell composition in our SLE cohort at a single-cell level. To do so, we performed an unbiased profiling of 9 peripheral blood mononuclear cell (PBMC) samples from 3 patients with SLE by cellular indexing of transcriptomes and epitopes by sequencing (CITE-Seq) analysis. PBMC samples collected on day 0 (baseline), day 1–2 after primary vaccination, and day 1–2 after secondary vaccination was used for the analysis. The CITE-Seq analysis combines highly multiplexed, surface protein marker detection (47 oligo-tagged antibodies including 4 isotype controls) and unbiased, single-cell transcriptomic profiling from 28,377 single cells. These data were then integrated with existing CITE-Seq data from 6 HC (15) (Figure 6, A and B). We identified 17 immune cell clusters from the integrated data sets (Figure 6C) (15).

We have recently demonstrated that the BNT162b2 vaccination in HC induces a substantially enhanced innate immune response after secondary vaccination. Using single-cell RNA-Seq analysis, we observed an increase in the frequency of a myeloid cell population termed C8 cluster (which expresses *CD14*, *VCAN*, *CD1C*, *FCGR1A*, and *CD274* mRNA or protein) 1 day after secondary vaccination, which is primarily composed of classical monocytes but also contains nonclassical monocytes and myeloid DC subsets and correlates with plasma IFN-γ levels (15). Integrating our published data set with that of the patients with SLE analyzed in this study, we identified the same population of cells expressing *CD274* and high levels of ISGs, including *WARS*, *GBP1*, *GBP5*, *IFI30*, *IFI35*, and *IFITM3*, and termed them “C8” for consistency (Figure 6, D and E, and Supplemental Figure 7). Interestingly, the C8 cluster was observed at a frequency of 1.8% of all monocytes and cDCs at baseline in patients with SLE, which was significantly higher (*P* = 4.2 × 10^–15^ by χ^2^ test, Figure 6, D and F) than observed in HC (0.038%). Furthermore, patients with SLE demonstrated a greater than 7-fold impairment in the induction of C8 cluster after secondary vaccination (Figure 6, D and F) relative to the induction in HC (6.9% versus 49.4%, *P* < 2.2 × 10^–16^ by χ^2^ test). To validate this in a larger dataset, we additionally analyzed 4 representative genes highly expressed in C8 cluster, including *GBP1*, *GBP5*, *ANKRD22*, and *CD274*, in the bulk RNA-Seq data from 18 patients with SLE and 32 HC as a proxy for C8 in all the participants. Our analysis displayed a similar pattern of increased baseline expression of these 4 C8 representative genes with reduced induction after vaccination in SLE-NR compared with HC (Supplemental Figure 8, A and B), further supporting an impairment of C8 myeloid induction in patients with SLE as compared with HC. Furthermore, the expression of these genes is highly correlated with plasma IFN-γ level (Spearman’s rank correlation coefficient = 0.79, *P* < 2.2 × 10^–16^, Supplemental Figure 8C).

## Discussion

This prospective study was designed to comprehensively assess the clinical features as well as the innate and adaptive immune responses in patients with SLE following 2-dose BNT162b2 mRNA vaccination using multiple high-dimensional technologies. Our study represents the first systems vaccinology analysis of the SARS-CoV-2 mRNA vaccine to our knowledge in this patient population. In addition, the present investigation was conducted in parallel with an HC cohort evaluated in the same manner (15), generating an unprecedented amount of data on the innate and adaptive immune responses in those with SLE in comparison with healthy individuals.

Recent data have shown that individuals with SLE exhibit diminished humoral responses to the SARS-CoV-2 vaccine relative to HC (18, 26). Results from our study are consistent with these findings, revealing decreased anti-Spike and anti-RBD antibody responses in patients with SLE following SARS-CoV-2 vaccination. Notably, the reduction in antibody responses was particularly prominent in participants receiving immunosuppressive medications. Current recommendations for withholding medications in individuals with AID subsequent to vaccination are based on expert consensus due to lack of data to support discontinuation regimens (27). Thus, these findings underscore the need for further studies assessing the potential benefits of temporary medication discontinuation during vaccination, as previously explored in the context of influenza vaccination and MTX (28).

The phenotypic and autoantibody heterogeneity observed among patients with SLE may lead to varying immune responses to SARS-CoV-2 vaccination. Indeed, stratification by autoantibody production revealed that all 6 patients with SLE with anti-Ro52 and anti-Ro60 IgG autoantibodies were NRs, and of 7 total NRs, only 1 did not have anti-Ro52 or anti-Ro60 autoantibodies. Prior studies have shown that patients with anti-Ro52 and anti-Ro60 antibodies have higher rates of leukopenia and increased production of CXCL10, potentially contributing to a lowered vaccine efficacy in this subgroup (29). Of note, 5 of the 6 patients with SLE with anti-Ro52 and anti-Ro60 autoantibodies were taking an immunosuppressive agent at the time of this study, an important confounder that could also explain these results. In either case, our data suggest that patients with SLE and preexisting Ro52 and Ro60 autoreactivity might be at higher risk of vaccine failure. Further studies of much larger cohorts are needed to differentiate between the role of preexisting anti–Ro IgG and immunosuppression in poor vaccine responses.

In addition to antibody responses, T cell responses play a pivotal role in providing protection against viral infections, contributing to long-term immunity. Moreover, T cells can confer cross-reactivity against multiple viral strains, as evidenced by a recent study that found an association between the HLA-B*15:01 allele and asymptomatic SARS-CoV-2 infection, implicating preexisting T cell immunity from prior exposure to HKU1-CoV and OC43-CoV as a putative mechanism for cross-reactive protection against SARS-CoV-2 (30). Persons with SLE have intrinsic quantitative and qualitative lymphocyte defects, rendering them more susceptible to viral infections (31). Consistent with these observations, prior studies have reported reduced IFN-γ release from T cells following SARS-CoV-2 vaccination in patients with SLE (18, 26). We rigorously assessed T cell responses in patients with SLE after BNT162b2 vaccination using spheromers specific for Spike protein epitopes bound to either HLA-A*02:01 or DRB*15:01 (24) to identify MHC Class I– or MHC Class II–restricted T cells and detected a consistently reduced induction of SARS-CoV-2 Spike–specific CD8^+^ and CD4^+^ T cells. Furthermore, in contrast to humoral responses, impaired T cell induction was also observed in patients with SLE not taking immunosuppressive medication. These effects may be due to intrinsic SLE-related impairments in T cell function (32, 33), highlighting the need for further studies aimed at enhancing T cell responses and optimizing vaccine efficacy in those with SLE.

Evaluation of SLE flares following mRNA vaccination is particularly important, given the reported vaccine hesitancy among persons with this condition. Concerns result from the possibility that autoantibodies against RNA-containing autoantigens, prevalent in patients with SLE, may not only dampen responses to an mRNA vaccine but may also promote flares driven by immune responses against ribonucleoproteins in immune complexes. Encouragingly, we did not observe an increase in autoantibodies or anti-cytokine antibodies after 2 vaccination doses in our SLE cohort. However, it is important to note that our study only assessed 51 autoantibodies and did not include some anti-phospholipid antibodies, which have been associated with prothrombotic events after COVID-19 infection (34). Although 4 participants experienced minor disease flares, there were no severe adverse events during the course of this study. Our results add to the substantial body of evidence confirming the safety of vaccination among patients with SLE (18, 26) and provide robust immunologic and clinical data to reduce vaccine hesitancy in this patient population.

Innate immunity plays a critical role in shaping adaptive immune responses after infection and vaccination. In particular, SARS-CoV-2 vaccination was shown to induce IFN-γ and CXCL10 signaling in HC, both of which positively correlate with antibody titers against SARS-CoV-2 Spike–RBD (35). SLE is characterized by chronic activation of type I IFN signaling in monocytes, the same pathway activated following mRNA vaccination in HC (36, 37). Consistent with this finding, bulk transcriptome analysis identified higher baseline levels of type I IFN signaling and CXCL10 in patients with SLE compared with HC. We further found that higher baseline type I IFN signaling in patients with SLE was associated with reduced vaccine-induced humoral responses. Our results align with prior studies demonstrating impaired responses to influenza and pneumococcal vaccine in patients with SLE, with higher IFN levels correlating with decreased neutralizing antibodies against vaccine antigens (38, 39). Collectively, these data suggest that chronic type I IFN activity adversely affects mRNA vaccine efficacy in patients with SLE.

Further underscoring the pivotal role of IFN signaling following vaccination, prior research has shown that BNT162b2 vaccination activates intermediate blood monocytes, increasing their frequency by 100-fold and upregulating IFN-response transcription factors (40). Another recent study of vaccine response in HC reported induction of a special monocyte population (C8 cluster) characterized by high levels of type I IFN gene expression after secondary vaccination (15). This population was not induced by SARS-CoV-2 infection, suggesting that the C8 monocyte cluster might play a functional role in resistance to SARS-CoV-2 infection. An alternative possibility is that the striking increase in the frequency of C8 monocytes following vaccination merely represents a response to higher circulating IFN-γ signaling in serum. Our findings demonstrate a higher baseline level of IFN-γ in the plasma as well as an increased frequency of the C8 monocyte cluster in patients with SLE compared with HC, suggesting that the C8 population may indeed represent an IFN-responsive monocyte cluster. We further found that induction of the C8 monocyte population in patients with SLE correlated with blood expression levels of IFN-responsive genes. However, further studies are needed to fully evaluate the role of these immune cells in vaccine protection.

Our study has several limitations, the first of which is that the generalizability of our findings is limited by the size of our SLE cohort. Second, although the study was designed to parallel the recent systems vaccinology study of HC (15), not all time points collected for analysis were identical for every patient. This variability limits some of the comparisons that can be made between the 2 cohorts. Finally, our time course was limited to 21 days after secondary vaccination; thus, it is unknown if tertiary (booster) vaccination might generate more productive immunologic responses.

Despite these limitations, our study substantially enhances our understanding of immunologic responses to SARS-CoV-2 mRNA vaccination in individuals with SLE, while also providing insights that may improve our understanding of mRNA vaccine responses in AID generally. Notably, our extensive immunologic data demonstrate impaired innate and adaptive immune responses in patients with SLE following BNT162b2 mRNA vaccination. Moreover, our findings suggest that high baseline IFN levels are associated with decreased vaccine efficacy, thus identifying potential biomarkers for future investigation. The observed effect of medications on antigen-specific neutralizing antibody responses further highlights an opportunity for new vaccination strategies to increase responsiveness. Finally, our comprehensive prospective study revealed no substantial adverse immunologic or clinical events after vaccination in our cohort, underscoring the safety of vaccination in people with SLE. Collectively, this study provides robust insights into how SLE affects mRNA vaccine responses and lays the groundwork for development of targeted vaccination strategies to improve efficacy in people with SLE and other connective tissue diseases.

## Methods

### Sex as a biological variable.

Only females were enrolled in this study, as 90% of individuals with SLE are female. Accordingly, the findings are highly relevant to females with SLE (the vast majority), and it is unknown if the findings will also apply to male patients with SLE.

### Clinical study design.

This prospective clinical study enrolled participants at Stanford Hospital and Clinics from March 2021 to May 2021. Eligible patients were 18 years or older, with a diagnosis of SLE according to the 2019 EULAR/ACR Classification Criteria (13). All patients underwent 2-dose vaccination against SARS-CoV-2 with the BNT152b2 vaccine at day 0 and day 21. Participants were evaluated at baseline, days 1 or 2, day 7, day 21, day 28, and day 42. The HC cohort was enrolled separately from December 2020 to February 2021 (15). Study assessments included: SLE activity measured with SLEDAI 2K score, which assigns points for 16 clinical and 8 lab items that signify organ involvement (41), adverse events measurements, changes in serological activity, autoantibody assay, anti-Spike antibodies, T cell quantification, and B lymphocyte subsets.

### Detection of SARS-CoV-2 and endemic coronavirus antibody titers.

Plasma samples from patients with SLE or HC were tested for levels of SARS-CoV-2 or endemic coronavirus antibodies using the MSD V-PLEX ECL-based assay (Plate 11 and Coronavirus Panel 2; Meso Scale Diagnostics), according to manufacturer’s instructions. Briefly, samples were diluted 1:5,000 in diluent and plated after blocking on the indicated multiplex antigen MSD plates. Detection was performed using MSD-provided IgG secondary, and signal was recorded on an MSD detection instrument according to manufacturer’s instructions. Data were analyzed using the MSD discovery workbench.

### ACE2 blocking assay.

Plasma samples from patients with SLE or HC were assessed for their ability to block the interaction between SARS-CoV-2 Wuhan Hu-1 Spike protein and ACE2 using an ECL detection system in 96-well plates (MSD V-PLEX SARS-CoV-2 Plate 11) and an MSD detection instrument according to the manufacturer’s instructions. Plasma samples were diluted 1:100. For a negative control, diluent without plasma was used to establish background signal levels, which were used to calculated percent inhibition. Data were analyzed using MSD discovery workbench.

### Neutralization assays.

Plasma samples were assessed for neutralizing antibody response using a SARS-CoV-2 pseudotyped lentivirus assay (42). Spike pseudotyped lentivirus was generated using the 5-plasmid system with mutations in the Spike for the following VOCs: WT, Beta (B.1.351), Delta (B.1.617.2), and Omicron (B.1.1.529). One day prior to infection, HeLa cells that overexpress ACE2 and TMPRSS2 were plated at 5,000 cells per well in 96-well, white-walled clear-bottom plates (Thermo Fisher Scientific). Viral dilutions for each VOC were made in DMEM (Corning), 10% FBS, L-glutamate, penicillin, streptomycin (all from GeminiBio), and 10 mM HEPES (Cytiva). Heat-inactivated plasma samples (diluted to achieve a final concentration of 1:1,250 in sterile DMEM, 10% FBS, L-glutamate, penicillin, streptomycin, and 10 mM HEPES) were incubated with Spike-pseudotyped lentivirus in the presence of polybrene (Sigma-Aldrich) at a final concentration of 5 mg/mL in all samples at 37°C for 1 hour. Heat-inactivated plasma samples were mixed with 50 mL of viral dilution to achieve a final volume of 100 mL in each well and a final plasma sample dilution of 1:1,250. After incubation, the plasma/virus mixture was transferred to the previously plated HeLa/ACE2/TMPRSS2 cells. Cells with the plasma/virus mixture were incubated at 37°C for 48 hours. Immediately prior to readout, the media were removed and replaced with a 100 mL 1:1 mixture of BriteLitePlus (PerkinElmer) and Dulbecco’s PBS (Thermo Fisher Scientific). Luminescence was measured using a BioTek Synergy HT (BioTek) or Tecan M200 microplate reader. Percent infectivity was determined by normalizing values from averaging cell-only wells (0% infectivity) and virus-cell (no plasma) wells (100% infectivity). Experiments were performed in technical duplicate in 2 separate experiments separated by time. Comparisons were made between HC and SLE cohorts using Kolmogorov-Smirnov’s test.

### Assembly of pMHC-spheromers.

A multimeric αβ T cell staining reagent (spheromere) reported recently by our group was used to analyze epitope-specific CD8^+^ and CD4^+^ T cell responses (43). MHC protein purification and peptide exchange were conducted as previously described (44, 45). The engineered maxi-ferritin scaffold was also purified as described previously (43) and used for spheromer assembly. In brief, the assembly was performed in 2 steps: (a) generation of a semisaturated streptavidin-pMHC_2_ (SAv-pMHC_2_) complex, and (b) conjugation of SAv-pMHC_2_ to the functionalized maxi-ferritin scaffold. SAv-pMHC_2_ was obtained by incubating 1 μM of the pMHC with 0.45 μM of SAv at 25°C for 30 minutes without agitation. Subsequently, the spheromer complex was assembled by incubating SAv-pMHC_2_ with the functionalized scaffold for 1 hour at room temperature with gentle rotation. The fluorophore-conjugated SAv was sourced from Invitrogen. For the simultaneous detection of multiple SARS-CoV-2 Spike epitopes using the spheromer technology, we adapted a combinatorial staining approach developed previously (46). Briefly, each peptide was assigned a unique fluorophore-barcode that allows the simultaneous detection of 2^n^-1 specificities (n represents the number of fluorophores). The relative concentrations for pMHC monomers associated with each fluorophore label (Alexa Fluor 647 [Thermo Fisher Scientific, S21374], eFluor 450 [Thermo Fisher Scientific, 48-4317-8], Brilliant Violet 711 [BioLegend, 405241], Brilliant Violet 785 [BioLegend, 405249], PE [Thermo Fisher Scientific, 12-4317-87], PE/Dazzle 594 [BioLegend, 405247], and PE/Cyanine7 [Thermo Fisher Scientific, 25-4317-82]) were experimentally determined.

### Bead-based autoantigen array construction and probing.

We created 2 different custom, bead-based antigen arrays. The cytokine array was composed of 55 cytokines, chemokines, growth factors, acute phase proteins, and cell surface proteins (Supplemental Table 2) and the traditional autoantigen array was composed of 51 commercial protein antigens associated with connective tissue diseases. Each array was constructed and used for probing as previously described with modifications (22). In short, antigens (Supplemental Table 3) were conjugated to uniquely barcoded carboxylated magnetic beads (MagPlex-C, Luminex Corp.). For each assay, the bead array was distributed into a 384-well plate (Greiner Bio-One) by transfer of 5 μL bead array per well. In total, 45 μL of diluted serum or plasma sample was transferred into the 384-well plate containing the bead array. Samples were incubated for 60 minutes on a shaker at room temperature. Beads were washed with 3 × 60 μL PBS-Tween on a plate washer (EL406, Biotek) and then incubated with 50 μL of 1:1,000 diluted R-phycoerythrin–conjugated (R-PE–conjugated) Fc-γ–specific goat anti–human IgG F(ab’)2 fragment (Jackson ImmunoResearch, 106-116-098) for 30 minutes. The plate was washed with 3 × 60 μL PBS-Tween and resuspended in 50 μL PBS-Tween prior to analysis using a FlexMap3D instrument (Luminex Corp.). Prototype human plasma samples derived from participants with AIDs with known reactivity patterns (e.g., Scl-70, centromere, SSA [Ro], SSB [La], whole histones, RNP, anti-IFN) were used as positive controls. Binding events were displayed as median fluorescence intensity (MFI). For normalization, MFI values for unconjugated, bare bead IDs were subtracted from MFI values for each antigen-conjugated bead ID for each sample. Criteria to define increases or decreases in autoantibody levels included (a) at least a 50% increase or decrease in MFI between time points; (b) an MFI of > 3,000 for subsequent time points or < 3,000 for preceding time points when defining increases or decreases, respectively; and (c) an MFI of > 3 SDs above the mean MFI of the HC at that time point.

### PBMC staining and flow cytometry.

PBMC staining and flow cytometry were performed as previously described. In brief, PBMCs were thawed in a water bath set at 37°C, and the cells were immediately transferred to warm RPMI media (Thermo Fisher Scientific) supplemented with 10% FBS (R&D Systems) and 100 U/mL of penicillin-streptomycin. After washing, the cells were filtered using a 70 μm cell strainer and rested for 1 hour at 37°C. T cells were enriched from PBMCs by negative selection using a FITC-conjugated antibody cocktail including anti-CD14 (clone HCD14, BioLegend), anti-CD19 (clone HIB19, BioLegend), anti-CD33 (clone HIM3-4, BioLegend), and anti-γδ TCR (331220, BioLegend) followed by magnetic bead depletion using anti-FITC microbeads (Miltenyi Biotec). The enriched T cells were washed and resuspended in FACS buffer for staining. All spheromer staining was performed for 1 hour after incubating the cells with Human TruStain FcX (BioLegend) for 15 minutes on ice. The spheromer were used at a monomeric concentration of ~100 nM and ~500 nM for the staining of CD8^+^ T cells and CD4^+^ T cells, respectively. Cells were subsequently stained with anti-CD19 (BV510, clone HIB19), anti-γδTCR (BV510, clone B1), anti-CD33 (BV510, clone HIM3-4), anti-CD3 (BioLegend, PE/Cyanine7, clone OKT3), anti-CD8 (BUV396, clone RPA-T8, BD Biosciences), anti-CD4 (BioLegend, BV785, clone RPA-T4), anti-CCR7 (BioLegend, PE/Dazzle 594, clone G043H7), and anti-CD45RA (BioLegend, BV711, clone HI100) and an amine-reactive viability stain (Live/dead fixable aqua dead cell stain kit; Invitrogen) for 30 minutes on ice. They were then washed, resuspended in FACS buffer, and acquired on a BD LSRII flow cytometer. The data were analyzed using FlowJo (v10) software.

### Plasma protein profiling using Olink Target 96 inflammation panel.

Cytokines in plasma were analyzed using Olink multiplex proximity extension assay (PEA) inflammation panel (Olink proteomics; www.olink.com) according to the manufacturer’s instructions. The PEA is a dual-recognition immunoassay, in which 2 matched antibodies labeled with unique DNA oligonucleotides simultaneously bind to a target protein in solution. This brings the 2 antibodies into proximity, allowing their DNA oligonucleotides to hybridize, serving as a template for a DNA polymerase-dependent extension step. This creates a double-stranded DNA “barcode” that is unique for the specific antigen and quantitatively proportional to the initial concentration of target protein. The hybridization and extension are immediately followed by PCR amplification, and the amplicon is then finally quantified by microfluidic quantitative PCR (qPCR) using Fluidigm BioMark HD system (Fluidigm).

Normalized Protein expression (NPX) values were used for downstream analysis. We used the limma (v3.54.2) R package to calculate fold changes both within group and between group (47). For within-group analysis, we used mixed-effects models to account for the repeated measures on the same patient across different time points. The *P* values from limma modeling were adjusted by the Benjamini-Hochberg method (FDR). The fold changes with FDR less than 20% are shown on the heatmaps.

### Sample preparation and RNA-Seq.

RNA extraction was carried out using Thermo Fisher Scientific’s MagMAX kit, specifically designed for PAXgene Blood RNA Tubes. Subsequently, sample quality control was conducted using the Agilent 2100 bioanalyzer, with a threshold set at RIN > 7 to identify suitable samples. Qualified RNA from each sample was then subjected to nonstranded RNA-Seq library preparation, which included globin depletion. The library preparation process involved mRNA fragmentation, followed by the generation of first-strand cDNA using random hexamer-primed reverse transcription. This was followed by second-strand cDNA synthesis and adapter ligation reactions. The resulting libraries were PCR-enriched and purified using Ampure XP beads, with library quantification performed using the Agilent Technologies 2100 bioanalyzer. The subsequent steps included heat denaturation and circularization of double-stranded PCR products, leading to the formation of single-stranded circular (ssCir) DNA libraries. These libraries were then amplified with phi29 to create DNA nanoballs (DNBs), each containing more than 300 copies of a single molecular entity. These DNBs were loaded onto a patterned nanoarray, and sequencing was carried out to generate paired-end 150 bp reads through sequenced by synthesis using the DNBSEQ-G400 platform.

### RNA-Seq analysis.

We used Trim Galore (v0.6.5) to trim Illumina adaptors from the raw fastq reads and removed reads that were too short after adaptor trimming (less than 20 nt). We then used Salmon (48) (v1.2.1) to obtain transcript-level expression based on human transcriptome sequences from GENCODE site (v32). Gene-level expression was summarized using Tximport (v1.16.0) (49). RNA-Seq data from the previous HC study were also processed using the same workflow, and the 2 RNA-Seq data sets were integrated using ComBat-Seq from the sva (v3.46.0) R package (50). The Voom (51) method was used to normalize the read count for linear modeling with limma (47) (v3.54.2), and fold changes were calculated both within group and between group. For within-group analysis, we used mixed-effects models to account for the repeated measures on the same patient across different time points.

### GSEA.

Genes with *P* ≤ 0.01 in any within-group or between-group comparison were selected and ranked by log_2_ fold change in each comparison, and they were then used as input in GSEA implemented in the fgsea R package (52). Enrichment was assessed with gene lists in blood transcriptomic modules (BTM) (53). The *P* values from GSEA were adjusted by the Benjamini-Hochberg method (FDR). The BTM terms with FDR less than 5% are shown on the heatmap. The ISG score was calculated as the geometric mean of 40 differentially expressed genes (FDR ≤ 0.05) in 6 BTM terms that are involved in antiviral interferon response (M111.0, M111.1, M75, M150, M127, M68).

### In silico cellular deconvolution using immunoStates.

We performed in silico cellular deconvolution of bulk RNA-Seq data using immunoStates (25) as a basis matrix with support vector regression to estimate proportions of immune cell subsets in each sample.

### Single-cell RNA-Seq.

CITE-Seq analysis of PBMCs was performed as previously described (54). In brief, live frozen PBMCs were thawed and washed twice with RPMI supplemented with 10% FBS and 20 μg/mL DNase I (Sigma Aldrich). Total PBMCs (1 million cells) were stained with a cocktail of TotalSeq-A antibodies (BioLegend) in PBS supplemented with 5% FBS, 2 mM EDTA and 5/mg/mL human IgG, washed twice with PBS supplemented with 5% FBS, and 2 mM EDTA, and resuspended in PBS supplemented with 1% BSA (Miltenyi Biotec), and 0.5/U/μL^−1^ RNase Inhibitor (Sigma Aldrich). Approximately 9,000 cells were targeted for each experiment. Cells were mixed with the reverse transcription mix and subjected to partitioning along with the Chromium gel-beads using the 10X Chromium system to generate the gel-bead in emulsions (GEMs) using the 3′V3 chemistry (10X Genomics). The reverse transcription reaction was conducted in the C1000 touch PCR instrument (Bio-Rad). Barcoded cDNA was extracted from the GEMs by post-GEM reverse transcription cleanup and amplified for 12 cycles. Before amplification, the cDNA amplification mix was spiked in with ADT additive primer (0.2 μM stock) to amplify the antibody barcodes. Amplified cDNA was subjected to 0.6× SPRI beads cleanup (Beckman, B23318). Amplified antibody barcodes were recovered from the supernatant and were processed to generate TotalSeq-A libraries as instructed by the manufacturer (BioLegend, TotalSeq-A antibodies with 10X Single Cell 3′ Reagent Kit v.3 3.1 protocol). The rest of the amplified cDNA was subjected to enzymatic fragmentation, end repair, A-tailing, adaptor ligation, and 10X-specific sample indexing as per manufacturer protocol. Libraries were quantified using Bioanalyzer (Agilent) analysis. 10X Genomics scRNA-Seq and TotalSeq-A libraries were pooled and sequenced on an Illumina HiSeq 4000 using the recommended sequencing read lengths of 28 bp (read 1), 8 bp (i7IndexRead), and 91 bp (read 2). CellRanger v.3.1.0 (10X Genomics) was used to demultiplex raw sequencing data and quantify transcript levels against the 10X Genomics GRCh38 reference v.3.0.0.

The single-cell RNA-Seq data were processed with Seurat (v4.0.5) (55). We removed cells with fewer than 200 or more than 8,000 detected genes, fewer than 400 or more than 80,000 mRNA reads, or more than 20% mitochondrial reads. This data set and the previous HC data set (15) were integrated using the Seurat integration workflow with reciprocal PCA algorithm. Afterward, clusters were identified with Seurat SNN graph construction on PCA embeddings after integration, followed by a Louvain community detection algorithm. The cell types were annotated using both canonical cell type markers and the cell type identities from the previous HC study. Cells were visualized in a low dimensional space using uniform manifold approximation and projection (UMAP).

### Figures.

All analysis was performed in R v4.2.2. Figures were generated using ggplot2 and Complexheatmap (56).

### Statistics.

Comparisons between SLE and HC cohorts for the neutralization assays and SARS-CoV-2 antibodies, and for ACE2-RBD blocking, were conducted using the Kolmogorov-Smirnov’s test. The association of Ro autoantibodies with vaccine response was calculated using the Fisher’s exact test. Comparison of SLE and HC cohorts for the CD8^+^ and CD4^+^ Spike–specific T cells throughout the vaccination series was conducted using the Mann-Whitney *U* test. The statistical approach for O-link cytokine analysis, GSEA, and scRNA-Seq are listed above in the corresponding methods sections (Plasma protein profiling using Olink Target 96 inflammation panel, GSEA, scRNA-Seq).

### Study approval.

This study was approved by the Stanford IRB (no. 60056), and all participants signed informed consent.

### Data availability.

The raw data have been deposited at GEO and are publicly available upon publication of the study, with accession nos. GSE260475, GSE260478, GSE250023, and GSE250024. Values for all data points in graphs are reported in the Supporting Data Values file. No original code was generated. All other code and scripts are available from the lead contact upon request. Further study-related patient data are available from the corresponding author upon request and will be deidentified before sharing.

## Author contributions

KYS, HZ, and LCZ authored the primary and subsequent drafts of the manuscript and compiled and analyzed key information and data. LCZ provided senior oversight of study design and study sample collection. LCZ, PJU, HTM, SDB, MMD, KCN, BP, and PK provided senior oversight of experiments and reagents. PSA, GS, FG, OFW, BL, EY, LWL, and AF conducted experiments and analyzed data. AE coordinated study sample collection. YC, MAL, and JL recruited patients for the study. All authors have read and approved the manuscript. KYS and HZ are co–first authors and are alphabetically ordered based on last name.

## Supplementary Material

Supplemental data

Supporting data values

## Figures and Tables

**Table 1:**
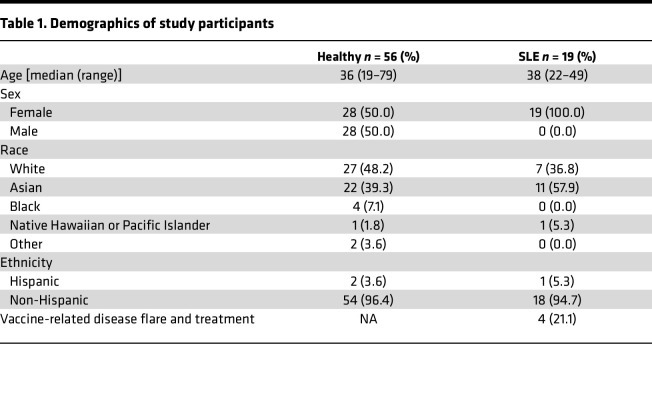
Demographics of study participants

## Version 1 03/08/2024

Electronic publication

## Version 2 06/21/2024

KCN's grant number was updated in the Acknowledgments section
