# Supplementary material for: Impaired innate and adaptive immune responses to BNT162b2 SARS-CoV-2 vaccination in systemic lupus erythematosus
Source: JCI Insight. 2024 Mar 8;9(5):e176556. doi: 10.1172/jci.insight.176556 (PMC10972586; doi:10.1172/jci.insight.176556)
Supplement: Supplemental data [file jciinsight-9-176556-s008.pdf]

Supplemental Conflict of Interest

KCN is an inventor or coinventor on the following:

Inventor, US Patent, entitled, "Methods and Assays for Detecting and Quantifying Pure Subpopulations of White Blood Cells in Immune System Disorders." Date filed 10/30/2018. Patent number 10114012.

Inventor, US Patent, entitled, "Granulocyte-based methods for detecting and monitoring immune system disorders." Patent Issued 2/13/18. Patent number 9891213.

Inventor, US Patent, entitled, "Mixed Allergen Compositions and Methods for Using the Same."

Dates filed
9/4/18 (patent number 10064936),
12/4/18 (patent number 10143742),
12/11/2018 (patent number 10149904),
1/1/2019 (patent number 10166286),
1/7/2020 (patent number 10525124),
1/7/20 (patent number 10525125),
6/30/20 (patent number 10695422),
5/18/21 (patent number 11007264),
10/19/2021 (patent number 11147871),
10/7/19 (patent number 1201706734S),
3/29/2021 (patent number 6859261),
8/15/17 (patent number 9731003),
12/14/2021 (patent number ZL201680017213.2).

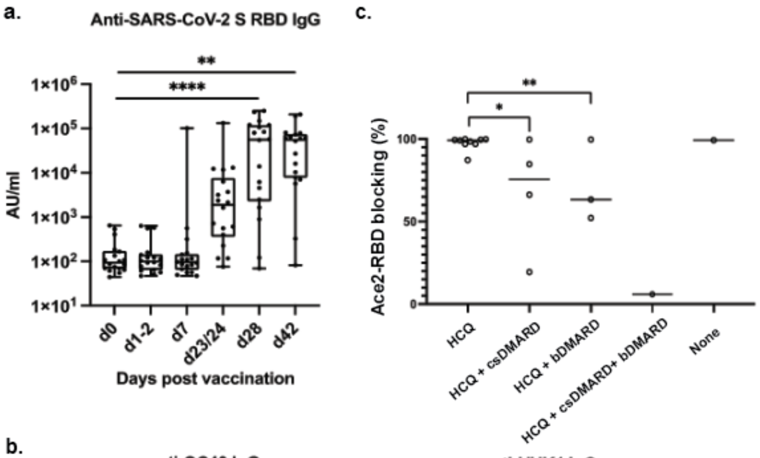

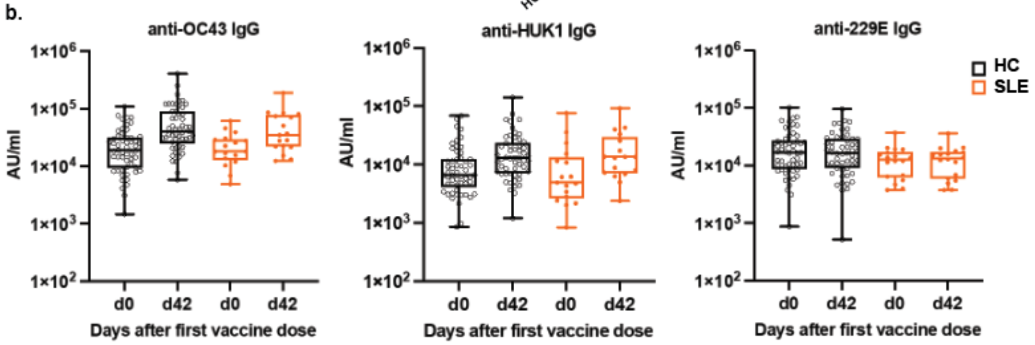

d.

	SLEDAI score	anti-Spike	anti-RBD	ACE2-RBD blocking	WT infectivity	delta infectivity	omicron infectivity
SLEDAI score		0.07 2	0.182	0.168	0.197	0.389	0.929
anti-Spike	0.072		4.94E-09	6.86E-08	5.75E-07	0.019	0.648
anti-RBD	0.182	4.94E-09		6.86E-08	1.09E-06	0.018	0.743
ACE2-RBD blocking	0.168	6.86E-08	6.86E-08		1.76E-06	0.009	0.763
WT infectivity	0.197	5.75E-07	1.09E-06	1.76E-06		0.002	0.831
delta infectivity	0.389	0.019	0.018	0.009	0.002		0.343
omicron infectivity	0.929	0.648	0.743	0.763	0.831	0.343	

Supplementary Figure 1. Antibody responses in SLE patients and HC after vaccination. (a) Time course of BNT162b2 elicited RBD antibody response in SLE patients. N=18 SLE (b) Antibody responses (day 42) against beta-coronavirus OC43, HKU1 and alpha-coronavirus 299E spike protein, as indicated, in fully vaccinated SLE patients. N=54 Healthy Controls (HC), 1N=8 SLE. (c) Comparison of RBD-ACE2 neutralization capacity to treatment modalities in the SLE vaccine cohort. Abbreviations: Hydroxychloroquine (HCq); conventional synthetic disease-modifying anti-rheumatic drug (csDMARD); biological disease-modifying anti-rheumatic drug (bDMARD) (d) p values corresponding to Spearman correlation analysis of anti-S, anti-RBD, RBD blocking, virus neutralization and clinical SLEDAI scores shown in Fig 1e.

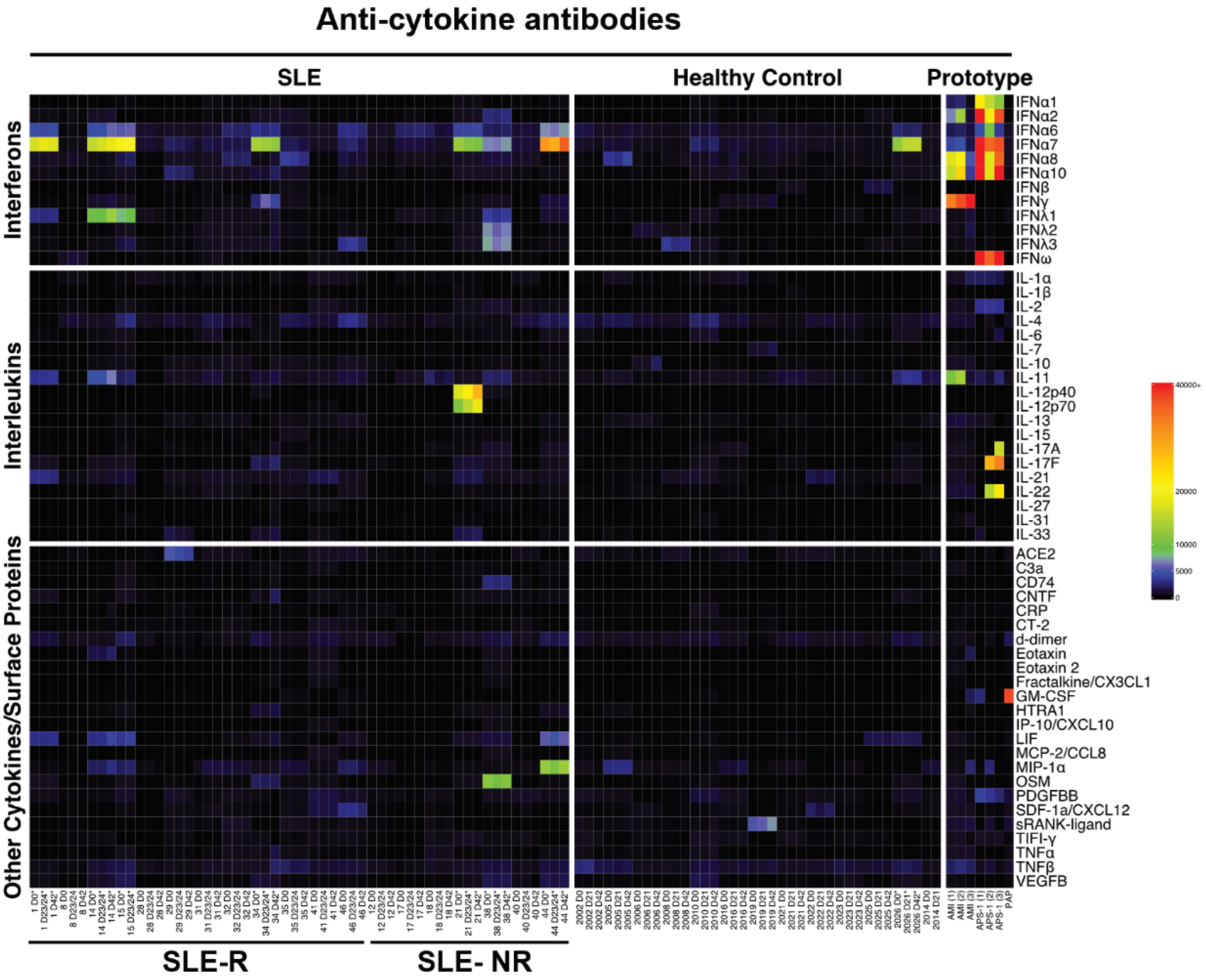

Supplementary Fig 2. BNT162b2 mRNA vaccination does not induce new anti-cytokine antibodies in SLE patients. Heat map of anti-cytokine antibodies (ACA) levels in serum of SLE patients (responders, R and non-responders, NR) and healthy controls on indicated days post vaccination was measured using a 56-plex micro-bead based cytokine array. MFI values are shown. N=13 healthy control, 19 SLE.

a.

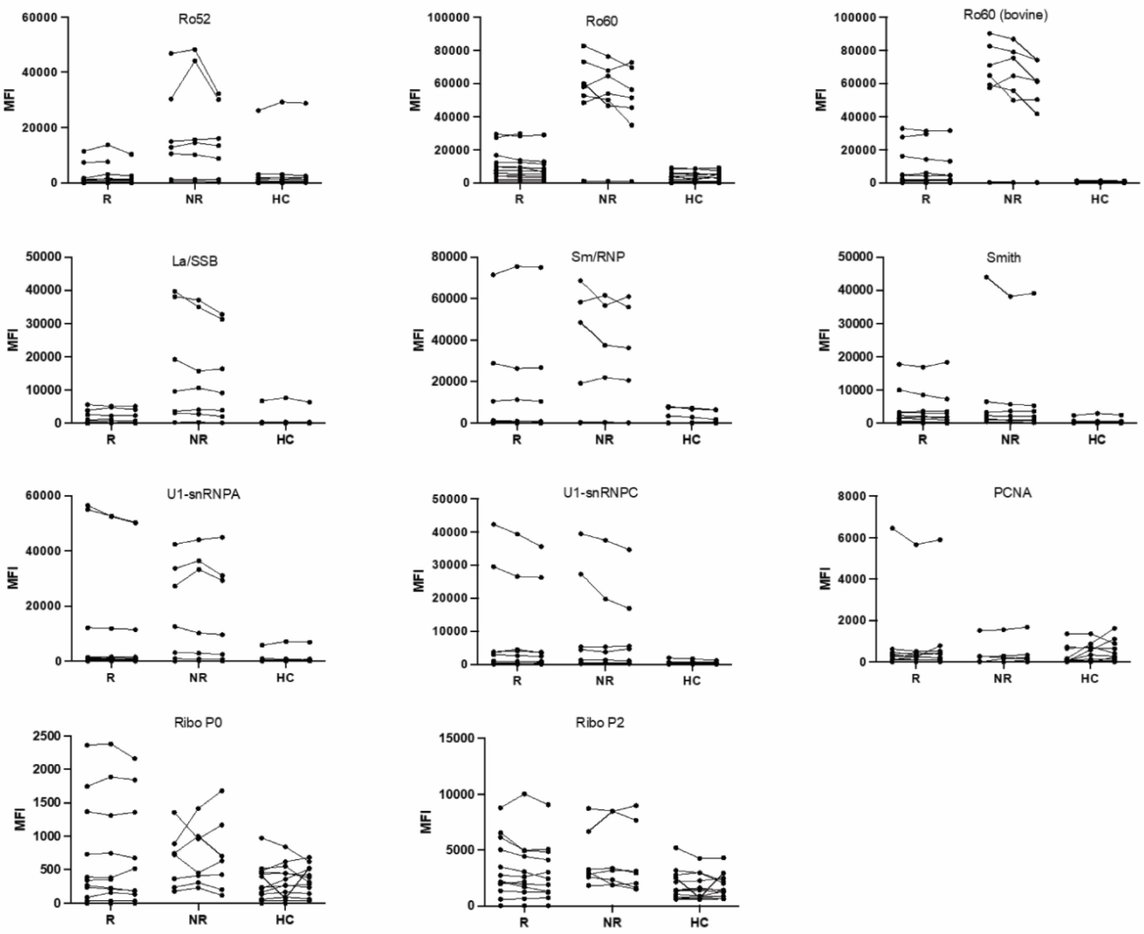

b.

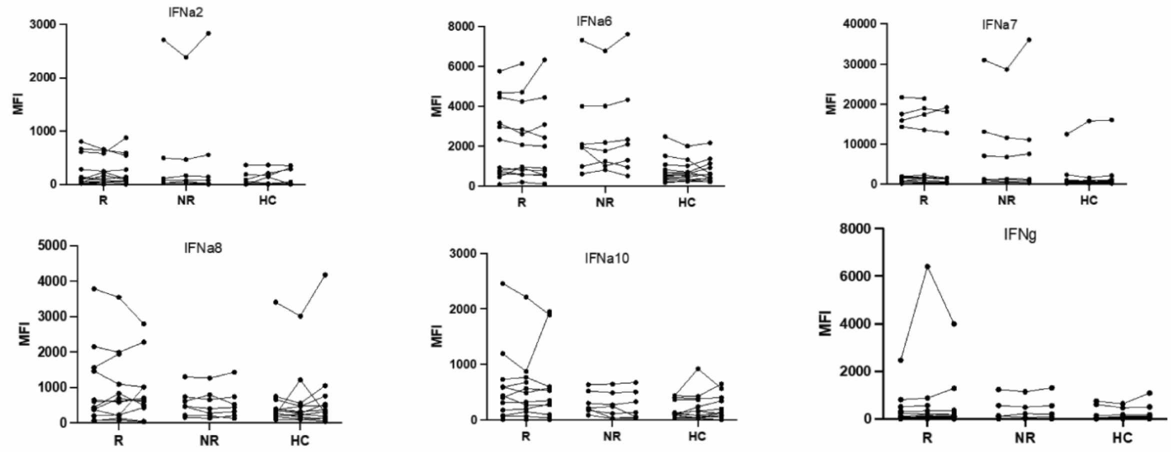

Supplementary Figure 3. Absence of new autoantibodies against traditional autoantigens (Supp 3a) or cytokines (Supp 3b). (a) Line plots showing SLE disease specific anti-nuclear antibodies (ANA) and (b) anti-interferon (IFN) antibody levels in serum of SLE patients (responders, R and non-responders, NR) on days 0, 23/24 and 42 post vaccination measured using the same micro-bead-based protein microarrays as in Figure 2 and Supplementary Figure 2. MFI values are shown. Only autoantibodies specific for interferon gamma increased transiently by more than 50% at D22/23 in one study participant (Supplementary Figure 3B, bottom right panel.

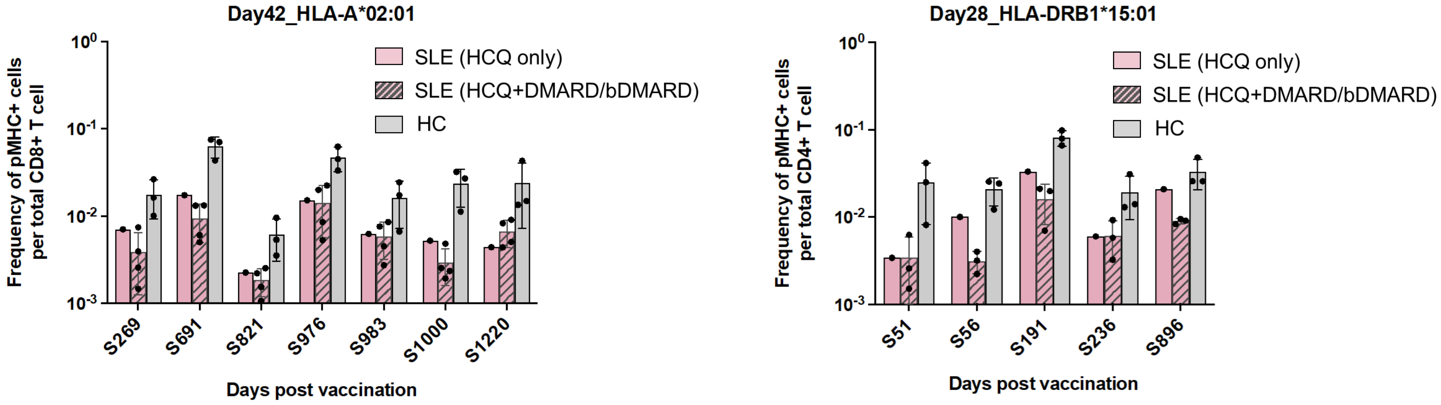

Supplementary Figure 4. Reduced frequencies of SARS-CoV-2 spike specific CD8+ and CD4+ T cells are seen in SLE even in the absence of immunosuppressive medication. (a) SARS-CoV-2 epitope specific CD8+ T cell frequencies (%) in PBMC isolated from HLA-A*02:01 SLE patients and healthy controls (HC) at day 42 post BNT162b2 administration was assessed using pMHC spheromer displaying SARS-CoV-2 epitopes. N=3 HC, N=6 SLE (2 taking only HCQ or chloroquine, 4 taking DMARDs/bDMARD) (b) SARS-CoV-2 epitope specific CD4+ T cell frequencies (%) in PBMC isolated from HLA-DRB1*15:01 SLE patients and HC at day 28 post BNT162b2 administration was measured using pMHC spheromer displaying SARS-CoV-2 epitopes. N=3 HC, N=4 SLE (1 taking HCQ only and 3 taking DMARDS/bDMARD). Data are presented as mean ± SEM.

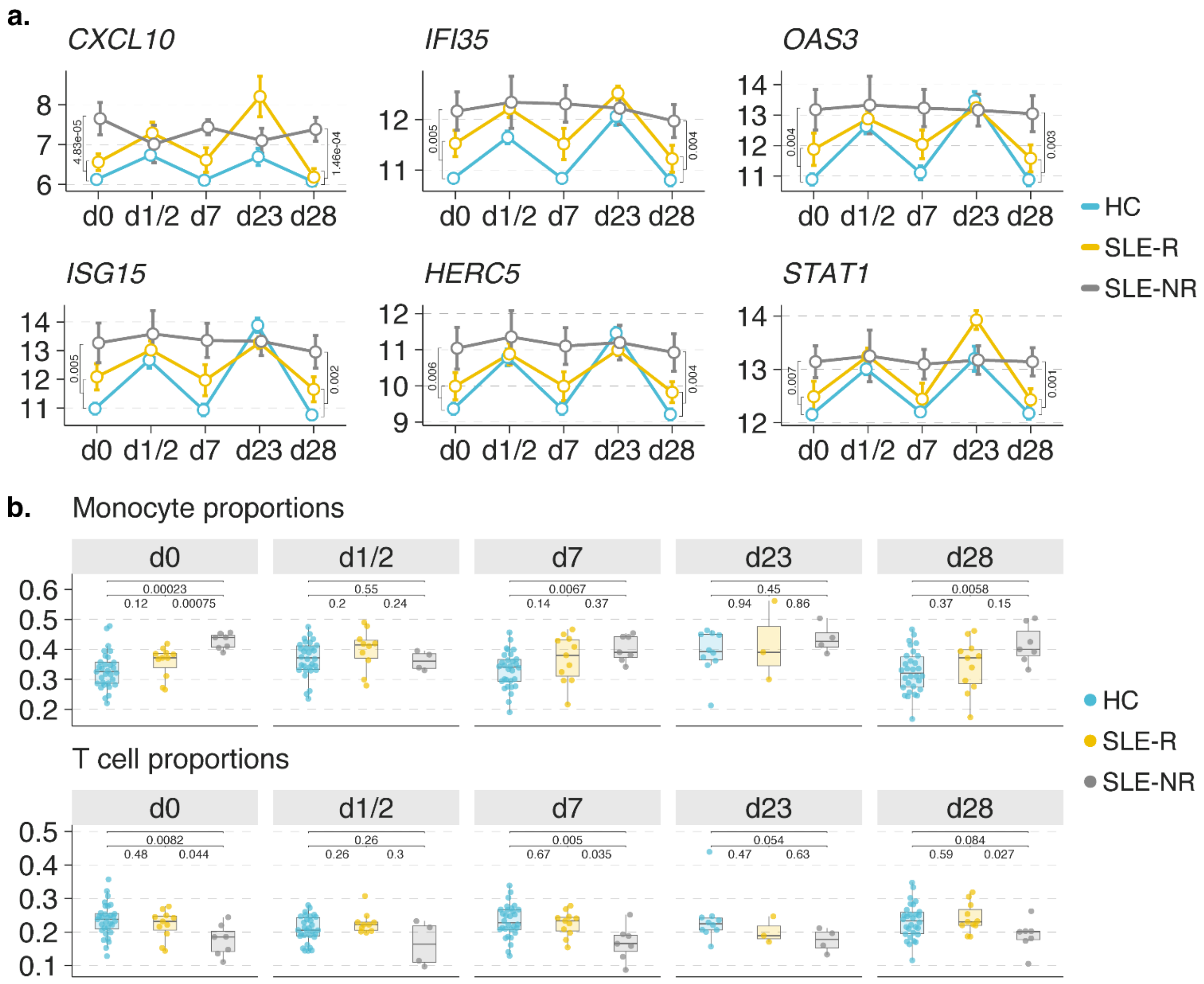

Supplementary Figure 5. Expression analysis and deconvolution of bulk RNA-sequencing data. (a) Line graphs depicting normalized gene expression levels of IFN responsive genes: *CXCL10, IFI35, OAS3, ISG15, HERC5* and *STAT1*. The p values for day 0 (SLE-NR: N = 7; SLE-R: N = 11; HC: N = 31) and day 28 (SLE-NR: N = 7; SLE-R: N = 11; HC: N = 30) were calcuated using one-sided Wilcoxon rank-sum test. (b) Estimated proportion of CD14+ monocytes and T-cells at each time point. P values calculated by Wilcoxon rank-sum test.

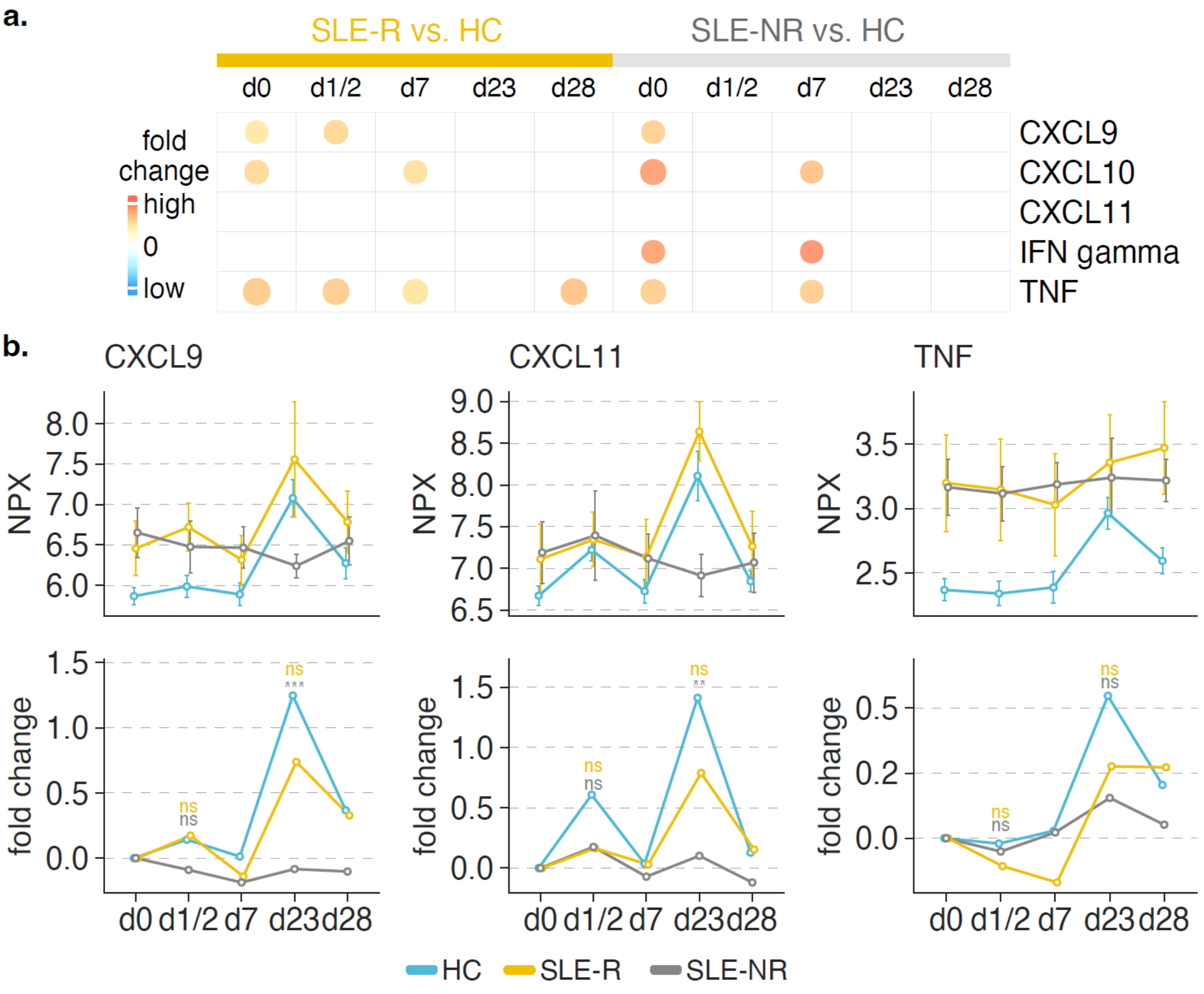

Supplementary Figure 6. Cytokine and chemokine responses in SLE patients and healthy controls (HC) after BNT162b2 vaccination. (a) Heatmap depicting fold-change in CXCL9, CXCL10, CXCL11, IFN-γ and TNF levels in SLE vaccine responders (SLE-R) and SLE vaccine nonresponders (SLE-NR) compared with HC, at interval time points (up to 28 days) after primary vacccination. The fold changes with FDR less than 20% were shown on the heatmap. (b) Normalized protein expression (NPX) and fold change (FC) of CXCL9, CXC11 and TNF as measured by Olink. The unadjusted p values were from between-group limma analysis after adjusting for baseline. * $p<0.05$, ** $p<0.01$, *** $p<0.001$. Samples numbers of each group: day 0 (SLE-NR: N = 7; SLE-R: N = 11; HC: N = 31); day 1/2 (SLE-NR: N = 5; SLE-R: N = 10; HC: N = 31); day 23 (SLE-NR: N = 4; SLE-R: N = 3; HC: N = 10)

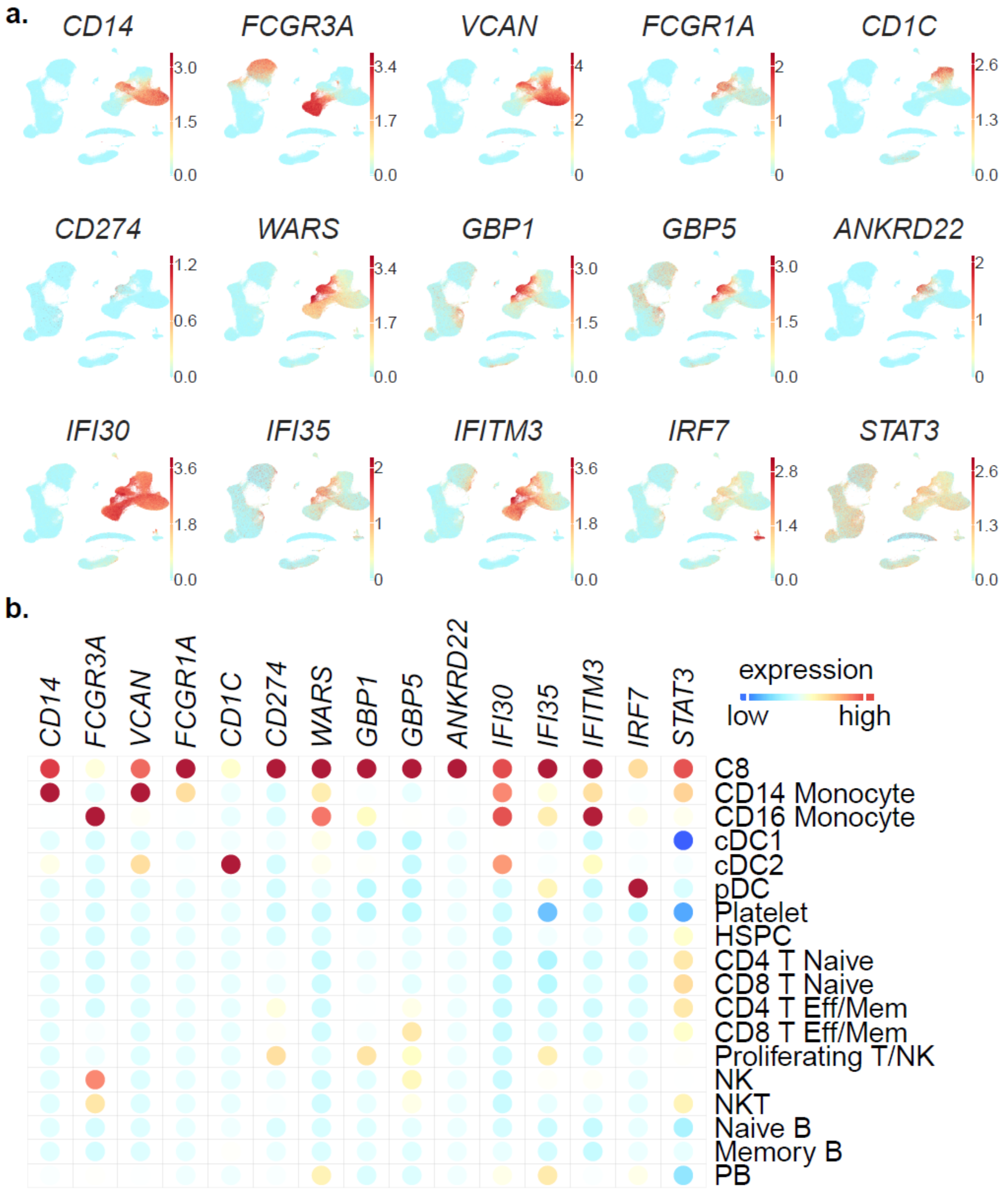

Supplementary Figure 7. Characterization of C8 cluster-related genes in single cell data. (a) Feature plots of myeloid cell markers and C8 cluster-related genes in SLE patients and healthy controls. (**b**) Heatmap of the mean expression of myeloid cell markers and genes highly expressed in C8 cluster. Row are the C8 cluster, and other remaining cells after separating out the C8 cells.

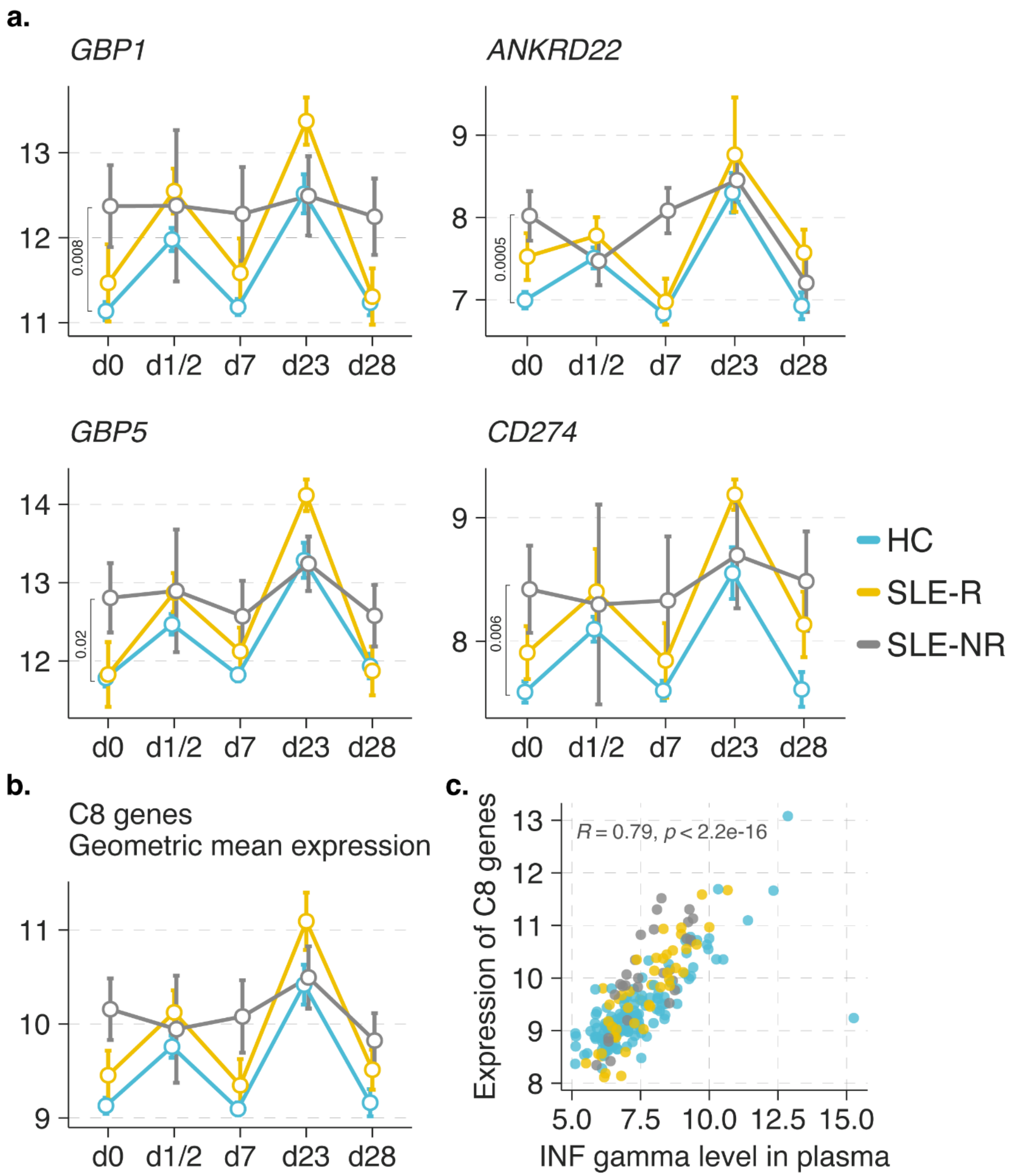

Supplementary Figure 8. Blood mRNA levels of C8 cluster representative genes in SLE and healthy control samples after vaccination profiled by bulk RNA-seq. (a) Line graphs of normalized gene expression values *GBP1, GBP5, ANKRD22,* and *CD274* across time points in SLE vaccine responders (SLE-R), SLE vaccine non-responders (SLE-NR) and healthy control (HC). The p values for comparing SLE-NR (N = 7) and HC (N = 31) at baseline were calcuated using one-sided Wilcoxon rank-sum test. (b) Line graphs of the geometric mean of *GBP1*, *GBP5*, *ANKRD22*, and *CD274* across time points in SLE patients and healthy controls. (c) Correlation plot of C8 gene expression and plasma IFN-γ levels.

Supplementary Table 1. Individual Demographics of Participants in Study.

Participant	Age	Sex	Autoantibody	Medication
0001	51	F		PLQ 300 mg daily
0008	30	F	Sm/RNP, U1-snRNPA, U1-snRNP C	Benlysta 200 mg subQ weekly, HCQ 200 mg daily
0012	45	F	Sm/RNP, Ro60, Ro60 bovine	Xeljanz 5 mg bid, Benlysta 200 mg subQ weekly, HCQ 400 mg daily, Prednisone 10mg daily, Colchicine 0.6mg daily
0014	42	F	PDC-E2, BP1	HCQ 200 mg every other day
0015	23	F	La/SSB, U11/U12	Cyclosporine 75 mg BID, Prednisone 5 mg daily, HCQ 200 mg daily, Cellcept 500 mg BID
0017	22	F	Sm/RNP, U1-snRNP A, Ro60, Ro60 bovine, La/SSB, U11/U12, SENP A	Benlysta 520 mg IV infusion monthly, HCQ 200 mg daily, Prednisone 5 mg daily
0018	32	F	U11/U12, Ro60, Ro60 bovine, Sm/RNP, Smith, U1-snRNP A, U1-snRNP C	Cellcept 1500 mg daily, HCQ 200 daily
0021	54	F	U11/U12, Ro60, Ro60 bovine	Cellcept 500 mg daily, HCQ 200 mg daily
0028	49	F	TPO	None
0029	38	F	RiboP1	HCQ 200 mg every 3rd day
0031	51	F	Scl70, Scl-70 truncated	HCQ 200 mg daily
0032	34	F	TPO, TG	HCQ 400 mg daily
0034	29	F		Imuran 100 mg daily, HCQ 200mg daily
0035	38	F	Sm/RNP, U1-snRNP, snRNP, Smith, BP1, TPO, TG	HCQ 400 mg daily
0038	48	F	Ro60, Ro60 bovine, La/SSB, Ribo P1	Quinacrine 100mg daily
0040	36	F	HLA-B27+	HCQ 300 mg daily, abatacept 750 mg monthly
0041	44	F		HCQ 300 mg daily
0044	25	F	Ro60, Ro60 bovine, La/SSB, Smith, U1-SnRNP, TPO, TG	Cellcept 1500 mg daily, HCQ 400 mg daily
0046	22	F		HCQ 200 mg daily

HCQ: hydroxychloroquine, BID: twice per day, IV: intravenous

Supplementary Table 2. Cytokine Array Content.

Bead ID	Antigen	Vendor	Catalog #
1	Bare Bead		
2	Human IgG from serum	Sigma	I4506
3	Anti-Human IgG Fc fragment specific	Jackson	109-005-008
4	Anti-Human IgG (H+L)	Jackson	109-005-003
5	Anti-Human IgG F(ab') fragment specific	Jackson	109-005-006
6	CD74	Prospec	PRO-1467
7	IFNλ2	Peprotech	300-02K
10	IL-1α	Prospec	CYT-253
13	IFNα1	Prospec	CYT-291
14	IFNα7	Prospec	CYT-196
16	IFNα10	Sino Biological	10349-H08H
18	IFNα8	Sino Biological	10347-H08H
19	IFNα6	Origene	TP760329
20	IL-2	Peprotech	200-02
21	IL-4	Peprotech	200-04
22	IL-13	Peprotech	200-13
23	IL-21	Peprotech	200-21
24	Fractalkine/CX3CL1	Peprotech	300-31
25	IP-10/CXCL10	Peprotech	300-12
26	IL-31	Prospec	CYT-625
27	IL-6	Prospec	CYT-098
28	MCP-2/CCL8	Peprotech	300-15
29	OSM	Peprotech	300-10
30	IL-11	Prospec	CYT-214
31	SDF-1a/CXCL12	Peprotech	300-28A
32	IL-27	Prospec	CYT-048
33	CNTF	Prospec	CYT-272
34	CT-2	Prospec	PRO-1578
38	GM-CSF	Peprotech	300-03
39	IFNα2	R&D	11101-2

40	IFNβ	Peprotech	300-02BC
41	IFNγ	Peprotech	300-02
43	IFNλ1	Peprotech	300-02L
44	IFNλ3	R&D	5259-IL-025/CF
45	IFNω	R&D	11395-1
46	IL-10	Peprotech	200-10
47	IL-12p40	Peprotech	200-12P40
48	IL-12p70	Peprotech	200-12
49	IL-15	Peprotech	200-15
50	IL-17F	Peprotech	200-25
51	IL-1β	Peprotech	200-01B
52	IL-22	Peprotech	200-22
55	TNFα	Peprotech	300-01A
56	TNFβ	Peprotech	300-01B
58	ACE2	Sino Biological	10108-H05H
59	Eotaxin	Peprotech	300-21
60	Eotaxin 2	Peprotech	300-33
62	IL-17A	Peprotech	200-17
63	IL-33	Peprotech	200-33
64	IL-7	Peprotech	200-07
65	MIP-1α	Peprotech	300-08
67	PDGFBB	Peprotech	100-14B
68	sRANK-ligand	Peprotech	310-01C
69	TIFIγ	Surmodics	A11001
70	CRP	Prospec	PRO-335
73	C3a	R&D	3677-C3-025
77	LIF	Peprotech	300-05
78	VEGFB	Peprotech	100-20B
79	HTRA1	R&D	2916-SE-020
80	d-dimer	LeeBio	200-13-0.1

Supplementary Table 3. Traditional Autoantigen Array Content.

Bead ID	Antigen	Vendor	Catalog #
1	Bare Bead		
2	Human IgG from serum	Sigma	I4506
3	Anti-Human IgG Fc fragment specific	Jackson	109-005-008
4	Anti-Human IgG (H+L)	Jackson	109-005-003
5	Anti-Human IgG F(ab') fragment specific	Jackson	109-005-006
6	Beta 2 Glycoprotein 1	Diarect	A14901
7	Myeloperoxidase	Diarect	A18501
10	Proteinase 3	Diarect	A18601
11	Histone 1	Immunovision	HIS-1001
12	Histone 2A and 4	Immunovision	HIS-1002
13	Histone 2B	Immunovision	HIS-1003
14	CENP B	Diarect	A12501
15	Histone 3	Immunovision	HIS-1004
16	Whole Histone	Immunovision	HIS-1000
17	GBM	Diarect	A16801
18	C1q	Biodesign	A90150H
19	BPI	Arotec	ATB01-02
24	Fibrillarin	Prospec	ENZ-566
29	La/SSB	Diarect	A12801
32	Ro 52/SSA	Diarect	A12701
38	CENP A	Diarect	A16901
39	EJ	Diarect	A11101
40	HSP 70	Stressgen	NSP-555
41	HSP 90	Stressgen	SPP-770
42	Intrinsic Factor	Diarect	A16701
43	JO1	Diarect	A12901
44	Ku, p70/p80	Diarect	A17301
45	LKM1	Diarect	A13501
46	MDA5	Diarect	A30001

47	MI-2	Diarect	A18101
48	PCNA	Diarect	A15401
49	PL-12	Diarect	A15701
50	PL-7	Diarect	A15601
51	PM/Scl-75	Diarect	A17001
52	Nucleolin	Diarect	A19701
53	Ribo P0	Diarect	A14101
55	PDC-E2	Diarect	A17901
56	Ribo P2	Diarect	A14301
57	SRP54	Diarect	A18401
58	PM/Scl-100	Diarect	A16001
59	POLR3H	Origene	TP310633
60	PDH	Sigma	P7032
61	Ro 60/SSA (bovine)	Diarect	A15501
62	Ro 60/SSA (recombinant)	Diarect	A17401
63	RPP14 (Th/To)	Origene	TP760291
65	Scl-70, full-length	Diarect	A12401
66	Scl-70, truncated	Diarect	A14501
67	Sm/RNP	Immunovision	SRC-3000
68	Smith	Immunovision	SMA-3000
70	Troponin I	Prospec	PRO-1269
73	TG	Diarect	A12201
75	MYH6	Origene	TP313673
76	TPO	Diarect	A12101
79	U1-snRNP A	Diarect	A13101
80	U1-snRNP C	Diarect	A13201
83	RPP25 (Th/To)	Origene	TP303538
